# Macrocolony Assays in the Rat of Allogeneic Y-P388 and W-256 Tumour Cells Injected Intravenously: Dependence of Colony Forming Efficiency on Age of Host and Immunity

**DOI:** 10.1038/bjc.1973.18

**Published:** 1973-02

**Authors:** H. A. S. van den Brenk, C. Sharpington, C. Orton

## Abstract

**Images:**


*Br. J. Cancer* (1973) **27**, 134.

### MACROCOLONY ASSAYS IN THE RAT OF ALLOGENEIC Y-P388 AND W-256 TUMOUR CELLS INJECTED INTRAVENOUSLY: DEPENDENCE OF COLONY FORMING EFFICIENCY ON AGE OF HOST AND IMMUNITY

H. A. S. VAN DEN BRENK, C. SHARPINGTON AND C. ORTON

*From the Richard Dimbleby Cancer Research Laboratory, St Thomas' Hospital, London SE1*

Received 24 August 1972. Accepted 23 October 1972

**Summary.—Two rapidly growing allogeneic tumours, sublines of Yoshida (Y-P388) and Walker (W-256) injected intravenously in single cell suspensions produced tumour macrocolonies in the lungs of rats within 7 days. Y-P388 produced similar but fewer colonies in the kidneys. Colony forming efficiency (CFE) in lung was high in weanling rats given either sublethal whole body irradiation (WBI) or a single dose of rabbit anti-rat lymphocytic serum (ALS) to suppress immunity. In immunologically intact weanlings CFE was much lower and many 7-day old colonies showed signs of regression. CFE for primary tumour cell challenges decreased rapidly and markedly with increase in age of host during the first 1–2 weeks after weaning. This resistance to growth of a primary challenge in lungs of older rats was not significantly reduced by WBI but was decreased by ALS. CFE of a secondary challenge of tumour cells injected intravenously in rats which had been previously immunized with heavily irradiated (HR) tumour cells was very low; it was not significantly increased by WBI but was moderately increased by ALS. In weanling rats given lethal (900 rad) WBI, 1 hour before intravenous injection of tumour cells, treatment with bone marrow (BM) cells derived from normal adult donors increased CFE, whereas BM (or spleen) cells from immunized donors decreased CFE. The results suggest that ALS and WBI not only increase tumour CFE by suppressing immunity to tumour growth but also " condition " host tissue (tumour bed) in such a way as to facilitate the survival, " take " and initial replication of grafted tumour cells before the rats recover from the immunosuppressive effects of these treatments.**

SINGLE tumour cells, prepared in suspension from certain transplantable ascites and solid tumours and injected intravenously, have been shown to form tumour macrocolonies in the lungs of mice and rats (Zeidman, McCutcheon and Coman, 1950; Baserga *et al.*, 1960; Williams and Till, 1966; Hill and Bush, 1960). The number of macrocolonies produced was found to be proportional to the number of cells injected, but colony forming efficiencies (CFE) reported in the literature varied considerably and were relatively low compared with plating efficiencies obtained *in vitro* (Williams and Till, 1966). A low CFE was also obtained in a syngeneic tumour–host system showing little evidence of immunological incompatibility (Hill and Bush, 1969), and in individual animals injected with the same number of cells colony numbers did not conform to a Poisson distribution.

This paper describes intravenous assays of two rapidly growing allogeneic tumours (Y-P388 sarcoma and W-256 carcinoma) which formed macrocolonies in the lungs of the rat within 7 days after injection and with high efficiencies. The effect of immunosuppressive agents on colony forming efficiency in hosts of different ages have been studied, as well as the effects of immunization and of treatment of inoculated rats with normal or sensitized bone marrow or spleen cells.

### MATERIALS AND METHODS

*Tumours.*—The 2 ascites tumours, a nitrogen mustard resistant subline (Y-P388) of the Yoshida sarcoma and the Walker (W-256) carcinoma, were used. These 2 tumours were obtained originally from Dr T. A. Connors, Chester Beatty Institute, London, and had been regularly passaged for some years in female Caworth Farm Strain (SPF) rats used in this laboratory exclusively. Both tumours produced rapidly growing solid, haemorrhagic growths (volume doubling time 16–24 hours) in muscle or in subcutaneous tissue, and they metastasized rapidly to regional lymph nodes and lungs; Y-P388 tumour metastasized regularly in all rats but only 50–70% of rats inoculated with W-256 developed growing metastases. Y-P388 cells measured 12·7 (10·8–13·5) $\mu$m mean diameter; W-256 cells were larger, measuring 14·8 (13·5–16·2) $\mu$m diameter. Single cell suspensions of either tumour inoculated intravenously in 3–4 week old weanling rats, produced blood red, raised macrocolonies (1–3 mm diameter) in the lungs after 6–7 days' growth. W-256 lung colonies were somewhat more discrete and larger than Y-P388 colonies (see Results). Intravenously inoculated W-256 cells rarely produced colonies in the kidneys. Rats inoculated with Y-P388 cells consistently developed kidney colonies, which were few in number but produced in proportion to the number of lung colonies.

*Whole body irradiation.*—A twin headed Mobiltron fitted with $^{60}Co$ sources was used for whole body irradiation (WBI). The technique has been described previously (van den Brenk, Moore and Sharpington, 1971*a*).

*Intravenous assay of tumour cells.*—All intravenous inoculations were standardized by injecting the required number of cells suspended in 0·5 ml ice cold Tyrode solution (pH 7·6) into a lateral tail vein of the rat. Weanling (3–4 week old) female rats of a specific pathogen-free (SPF) derived colony of the Caworth Farm Strain were used for most assays. They were exposed to 570 rad (WBI) less than 24 hours preceding inoculation, or were given 0·5 ml rabbit anti-rat antilymphocytic serum (ALS) intravenously on one or more successive days to suppress immunity. ALS serum was supplied by Burroughs Wellcome Ltd and had been prepared using rat thymocytes. The serum was not exposed to rat erythrocytes or other normal cells before use, in order to preserve maximum immunosuppressive potency. The first dose was given intravenously 1 day preceding inoculation or on the day of inoculation. Repeated doses of ALS caused a high incidence of renal damage as reported by others (see Guttmann *et al.*, 1967) and 20–40% of the rats died within 2 weeks. Rats inoculated with tumour were deeply anaesthetized 7 days after inoculation, exsanguinated and the abdomen and thorax were opened widely. The total number of macroscopically visible tumour colonies present on the external surfaces of both lungs was counted and a similar count was made for both kidneys in each rat. The thymus and spleen were usually removed and weighed. A group of not less than 6 rats was used for each point of an assay. The donor tumour cells were provided by freshly harvested ascites fluid which was diluted and counted as described previously (van den Brenk *et al.*, 1971*a*). To study the effect of age of recipient on colony forming efficiency, both male and female rats, aged from 28 to 63 days on the day of sacrifice, were used.

*HR tumour cells.*—These were prepared as described previously by exposing freshly harvested heparinized tumour ascites fluid in plastic dishes to a single dose of 6000 rad to destroy cell proliferative potential and clonogenicity. Intravenous injection of $10^6$ cells of these irradiated cells produced an average of $<1$ lung colony per rat. HR cells were used to increase immunity to tumour growth in rats by injecting the animals intramuscularly with $\sim 10^7$ HR cells twice weekly for 3 weeks.

*Colony assays.*—The mean number of lung colonies ($N_L$) or kidney colonies ($N_K$) present 7 days after inoculation was plotted as a function of the number of intravenously inoculated cells ($N$). It was found that if $N$ exceeded $10^4$ cells, $N_L$ was too high ($>200$) in immunologically suppressed weanling recipients to count colonies with sufficient accuracy since many colonies had become confluent. The presence of a large number of lung colonies also caused marked increases in lung weight due to tumour growth which induced focal haemorrhage and oedema. Colony forming efficiency was defined as the mean number of macrocolonies present on the surfaces of lungs or kidneys which was produced for each cell inoculated.

*Spleen and bone marrow cells and macrophages.*—Suspensions of spleen and bone marrow cells were prepared from spleens or from femoral and tibial bone marrow plugs respectively, which had been removed either from unimmunized or immunized donor rats. These cell suspensions were prepared in appropriate dilutions in heparinized ice-cold Tyrode solution in the usual way and were used to treat rats which had been inoculated with tumour cells intravenously. Peritoneal macrophages were harvested from rats 5 days after injecting 5 ml of 6% Na caseinate intraperitoneally (Patterson, Pisano and Di Luzio, 1970).

*Splenectomy.*—Under pentobarbitone sodium anaesthesia, the spleen was removed through a left subcostal incision and the wound approximated in layers with sutures and metal clips. Rats were inoculated with tumour cells 2–3 days after operation.

*Histological studies.*—Formalin fixed tissues were embedded in paraffin; 5–7 $\mu$m thick sections were cut and stained by haematoxylin and eosin or the van Gieson technique. The Unna–Pappenheim and acridine orange methods for staining nucleic acids were used to assist in the identification of plasma cells in tissue sections.

*$ED_{50}$ for tumours.*—The 2 tumours were inoculated intramuscularly in the gastrocnemius in 0·1 ml volumes containing from 5 to 5 $\times$ $10^6$ tumour cells. A group of 5–6 rats which were immunologically intact, immunologically attenuated (570 rad WBI) or which had been immunized ($10^7$ HR tumour cells twice weekly for 3 weeks) respectively were used for each cell dose inoculated. The number of cells required to induce a palpable growing tumour within 4 weeks in 50% of rats ($ED_{50}$ value) was calculated.

### RESULTS

### 1. *$ED_{50}$ values for intramuscular inoculation of Y-P388 and W-256 tumours*

Whereas a primary challenge of only a few (<10) W-256 cells was required to induce progressive growth in immunologically intact rats, $\sim 5 \times 10^3$ Y-P388 cells were required (Table I). Suppression of immunity by sublethal WBI (or ALS) lowered $ED_{50}$ values for the 2 tumours to $\sim$5–10 cells. Immunization raised values to $10^5$–$10^6$ cells. $ED_{50}$ values for the 2 tumours in muscle did not change significantly with increase in age of host. Indeed, the rate of growth of these tumours in muscle had been found to increase with age, a finding which will be reported in further detail in relation to effect of age on relative growth rates of primary Y-P388 tumour and its metastases.

TABLE I.—*Changes in $ED_{50}$ Values for Y-P388 and W-256 Tumour Inoculations* in muscle *of 6-week old Female Recipient Rats due to Immunological Suppression (WBI 570 rad or treatment with ALS) and Immunization ($10^7$ HR cells injected Twice Weekly for 3 weeks or Growth of Intact Cells as Primary Challenge)*

| Treatment | $ED_{50}$ (number of cells) | |
|---|---|---|
| | Y-P388 | W256 |
| Nil | $\sim 5 \times 10^3$ | < 10 |
| WBI | < 10 | < 5 |
| ALS | < 5 | < 5 |
| HR cells | $\sim 5 \times 10^5$ | $\sim 10^5$ |
| Growth of tumour* | $> 10^6$ | $> 10^6$ |

* $ED_{50}$ for second challenge in rats with 7-day old growing solid tumour induced by primary challenge ($10^5$ cells).

### 2. *Tumour macrocolony formation in lungs and kidneys*

In weanling (3–4 week old) female rats inoculated intravenously with either Y-P388 or W-256 tumour cells, lung and kidney colony counts ($N_L$, $N_K$) obtained have been plotted as functions of number of cells injected ($N$) on a log-log scale (Fig. 1). The relationships are essentially linear, such that

$$N_L = kN^{\theta}$$

where $\theta$ and k are constants for Y-P388 tumour cells; the value of exponent $\theta$ approximates to 0·7 for both lung and kidney colony production, and in both unirradiated and irradiated rats. W-256 produced no kidney colonies, even after injecting $10^4$ or more cells which caused confluent and consolidative growth of

FIG. 1.—Number of macrocolonies present 7 days after inoculation in lungs ($N_L$) and kidneys ($N_K$) of irradiated (closed symbols) and unirradiated (open symbols) weanling rats plotted as a function of number of Y-P388 or W-256 tumour cells ($N$) injected intravenously. Each point represents mean ($\pm$SE) for a group of 6–8 rats.

tumour in the lungs and considerable increases (two–three-fold) in lung weight. As a result, a proportion of such rats died within 7 days. For W-256, $\theta$ was higher (0·93) and was not significantly different for unirradiated and immunosuppressed recipients.

Less efficient colony production by both tumours occurred in immunologically intact rats. The number of Y-P388 cells injected had to be increased by factors of 0·8 and 1·8 to increase $N_L$ and $N_K$ respectively to the same values as were obtained in irradiated rats; for W-256, an 0·7 increase in log $N$ was required in unirradiated rats to raise lung colony production to that in irradiated rats.

Y-P388 macrocolonies in kidney had the same appearances and were similar in size to those in lung but were fewer in number ($N_K \simeq 0{\cdot}1\ N_L$ in irradiated rats and $N_K \simeq 0{\cdot}02\, N_L$ in immunologically intact rats). Colony production for W-256 increased linearly with increase in the number of cells injected, since $\theta$ (W-256) $= 0{\cdot}93$ and approximated to unity but the rates of increase in $N_L$ and $N_K$ for Y-P388 ($\theta = 0{\cdot}72$) were not linear. The greater proportion of Y-P388 cells than the larger W-256 cells, which escape arrest in the lungs, may help to account for the lower values of $\theta$ obtained for Y-P388 tumour cells.

Besides increasing $N_L$ and $N_K$, immunosuppressive treatments also caused Y-P388 and W-256 lung colonies to differ in size and appearance. In irradiated recipients, the colonies were larger and more uniform in size, were redder due to the presence of more blood and their growth caused greater increases in lung weight. In unirradiated recipients, lung colonies (particularly Y-P388 colonies) were paler and browner in colour and flattened. These signs of regression of growth were seen as early as 7 days after inoculation of weanling rats. The lung tissues surrounding regressing colonies were frequently depressed, in the form of a moat around each colony, and caused umbilication (Fig. 2). More marked signs of tumour regression occurred in older rats and particularly if colonies were allowed to grow for longer than 7 days. Further colony regression gave rise to pitting of the pleural surface due to focal atelectasis, with overlying small adherent plaques of fibrinous exudate and later still complete regression gave rise to discrete white scars, pinpoint in size (Fig. 2).

In irradiated weanling rats tumour colony counts were slightly lower than in rats treated with ALS. A few experiments in which rats received WBI combined with ALS, $N_L$ and $N_K$ for Y-P388 were slightly raised but this treatment proved highly lethal and caused 20–30% of rats to die within 7 days. Y-P388 and W-256 colonies in lungs differed in their histological appearances (Fig. 2). The latter were epithelioid in structure, more discrete and there was less infiltration of adjacent lung tissues by tumour. Special stains were used for plasma cells and india ink was injected intravenously to label macrophages intravitally. Neither growing nor regressing lung tumour colonies showed significant evidence of infiltration by lymphocytes, plasma cells or macrophages. More tumour cells in regressing colonies showed pyknotic and lytic changes but most tumour cells appeared viable on histological grounds and dividing tumour cells (mitosis) were usually seen. The free blood (haemorrhage) had largely disappeared in regressing colonies, and progressive reparative fibrosis was the principal change shown by such colonies in lung and kidney.

It was difficult to lay down strict criteria which defined clonogenicity of tumour in terms of macrocolony formation, since colonies differed in size. Even if clear evidence of initial take and growth of inoculated cells could be deduced by the presence of colonies, regressive changes caused alterations in both macroscopic and microscopic appearances. In the present study a macrocolony was arbitrarily defined as a discrete, *raised* colony, 1 mm or more in diameter, visible to the naked eye on the pleural surface. Any evidence of regression was ignored except if residual pitted and scarred lesions were present, which were not scored. Lung colony counts were made in 2 groups of 10 female and 10 male 32-day old rats (litter maters obtained from 4 litters born on the same day), which had been given 570 rad WBI and inoculated with $5 \times 10^2$ W-256 cells. There was no significant difference. The pooled data for $N_L$ in both sexes (Fig. 3) did not show a Poisson distribution (see Discussion). Results in Fig. 3 show that $N_L$ was $73 \pm 9$ in females and $71 \pm 9$ in males. This data pooled for the male and female rats used in this experiment gave a mean value $N_L = 72$, *i.e.* a colony forming efficiency of 0·14. This value is high compared with a maximum efficiency of 0·03 obtained by Hill and Bush (1969) for

FIG. 2.—Appearances of W-256 and Y-P388 tumour colonies in lungs of rats 7 days after intravenous injection of tumour cells. A. Unfixed lungs and kidneys of 6-week old rats injected with $10^4$ Y-P388 cells: *left*, after rats had been immunized with HR cells (see text) showing no colonies; *centre*, primary challenge in unirradiated rats, showing small pale regressing colonies in lungs; *right*, actively haemorrhagic colonies in lungs and kidneys of rats given 570 rad WBI 24 hours before inoculation. B. W-256 colonies on surface of formalin fixed lung from unirradiated rat; many colonies show regression causing umbilication (U) and the adherence of fibrinoid material (F). C. Histological appearances of W-256 colonies ($\times 30$). D. Histological appearances of Y-P388 colonies ($\times 30$).

Fig. 3.—Lung colony counts obtained in 2 groups of 10 female and 10 male 32-day old rats given 570 rad WBI before inoculating 5 × 10² W-256 cells.

a syngeneic system in the mouse, obtained by adding HR cells in excess to the inoculum which caused $N_L$ to increase twenty–thirty-fold.

Despite the high colony forming efficiency, the data in Fig. 3 show that the variance greatly exceeded the mean. Similar high variances were obtained when ALS was used instead of whole body irradiation to suppress immunity or when certain other procedures were used to increase values of $N_L$, namely, by adding HR cells to the inoculum, locally irradiating the lungs before the inoculation of tumour cells or combining WBI with steroid therapy (see below).

3. *Effect of age of recipient on colony production*

One hundred and twenty 26–30 day old female rats were randomized into 16 groups (6–8 rats per group) at the onset of an experiment designed to determine the effect of age of recipient on the production of lung colonies by intravenously injected Y-P388 tumour cells. Eight groups received 570 rad WBI, <24 hours preceding the injection of tumour cells; the other 8 groups were not irradiated. At 4 different ages (3–8 weeks) all rats in an unirradiated and an irradiated group were injected with $5 \times 10^2$ Y-P388 cells per 100 g body weight and 2 such further groups received $5 \times 10^3$ cells per 100 g body weight. All rats were killed 7 days after inoculation when mean rat body weights ranged from 85 to 205 g (corresponding to a 5-week increment in age of rats). $N_L$ and $N_K$ in both unirradiated and irradiated rats decreased rapidly with increase in age, particularly so during the fifth to sixth week (Fig. 4).

W-256 lung macrocolony production in irradiated rats was similarly dependent on age and not affected by sex (Fig. 5). In this experiment, 108 female and 90 male rats were given sublethal WBI and injected with $5 \times 10^2$ W-256 cells. Colony formation decreased even more rapidly (approximately ten-fold) during the sixth week of post-natal life. Resistance to clonogenicity of tumour in the lungs with age could not be attributed simply to increase in body weight (and increased lung volume) since males grew considerably faster than females during the course of the experiment (see Fig. 5).

In this strain of SPF rats, weight of most organs ($w$), including that of thymus, can be expressed in terms of body weight ($W$) as an allometric function (van den Brenk, Sparrow and Moore, 1969), given by the equation of Huxley (1932):

$$w = \alpha W^{\beta}$$

where $\alpha$ and $\beta$ are constants. In females, the thymus shows positive growth ($\beta = +1{\cdot}33$) until about the sixth week (130 g body weight) when it commences to regress and causes growth (in an allometric sense) to become negative, ($\beta = -1{\cdot}43$), with further ageing. In males, regression of the thymus is delayed until about the eighth week of age. Consequently in both sexes the age of onset of thymic regression did not correlate with the development of resistance to take and growth of tumour cells in the

FIG. 4.—Numbers of tumour macrocolonies produced in lungs ($N_L$) and kidneys ($N_K$) of female rats of different ages, 7 days after 5 × $10^2$ or 5 × $10^3$ Y-P388 cells/100 g body weight had been injected intravenously; unirradiated rats (open symbols), after 570 rad WBI (closed symbols), 6–8 rats per point.

FIG. 5.—Tumour colonies produced in lungs of male and female rats of different ages 7 days after intravenous injection of 5 × $10^2$ W-256 cells (upper figure). Lower figures show corresponding changes in mean final body weight, and in spleen and thymus weights (open symbols used for males, closed symbols for females). In lower figures the small symbols and interrupted lines represent organ weights corresponding to mean final body weight in untreated control rats; in left figure the body weight/age curves for normal males (M) and females (F) of this strain of rats are shown as uninterrupted lines. In all 4 graphs the larger circular symbols are for rats given 570 rad WBI, square symbols are for rats given a single i.v. injection (0·5 ml) of ALS before tumour inoculation, and the triangular symbols are for inoculation of rats which had received neither irradiation nor ALS (6 rats per point).

lungs; nor were allometric, and hyperplastic, increases in weight of spleen related to susceptibility to tumour growth (see below).

The results in Fig. 5 also show that unirradiated, recently weaned rats were much more resistant to growth of tumour in lungs ($N_L = 19 \pm 5$) than irradiated rats of the same age ($N_L = 91 \pm 10$).

When weanling rats were similarly irradiated but not injected with tumour cells until 14 days later (when 40 days old) $N_L$ was significantly higher than in 40-day old rats irradiated immediately preceding injection of the same number of W-256 cells (Table II), although the value obtained ($N_L = 57 \pm 13$; $N = 10^3$ cells) was lower than in recently weaned rats irradiated immediately before injection of tumour cells (see Fig. 1). Much of this decrease could be accounted for by the presence in the older rats of " microcolonies " which were not scored. Fourteen days after irradiation the weight of spleen in inoculated rats had recovered and increased to double that of normal rats of the same body weight, but the thymus remained ~40% lower in weight than normal.

4. *Arrest of body growth*

A further experiment was made to determine whether increase in body weight *per se* could account for resistance to tumour growth in lungs with increase in age of rats. The growth of 5-week old rats was stunted and their body weight reduced to that of 4-week old rats by restricting the dietary intake for 7 days to tapioca (supplemented with vitamins) with water *ad libitum*. This caused a mean body weight loss of 3·4 g per day compared with a similar rate of gain in weight on a normal diet. After 7 days on restricted diet the rats were given WBI and inoculated with $2 \times 10^2$ W-256 tumour cells and replaced on a normal diet. This caused mean rat growth rate during tumour growth (5 g per day) to exceed normal (~3·5 g per day). However, colony forming efficiency was only 0·012 and similar to the value 0·014 obtained in unstarved rats of the same age (49 days), whereas much higher values of 0·155 and 0·180 were obtained in 35- and 42-day old rats respectively (Table III). Consequently, resistance to lung colony growth of tumour is associated with ageing and is not dependent on body weight.

5. *Effects of treatment with ALS serum*

In older (56-day old) rats given a single intravenous injection of 0·5 ml of ALS, the incidence of lung tumour colonies was comparable to that in weanling rats given ALS or WBI (Fig. 5) *i.e. in both sexes ALS but not irradiation had reversed resistance to clonogenic growth of a primary challenge of W-256 and Y-P388 tumours in the lungs, which develops with increase in age of rat whereassu blethal whole body irradiation failed to do so.* Thus, in 56-day old rats injected with $5 \times 10^2$ W-256 cells, colony forming efficiency increased from 0·009 (after WBI) to 0·234 (after ALS), *i.e.* approximately

TABLE II.—*Effect of WBI (570 rad) given Immediately or 14 Days Before Intravenous Inoculation of Female Rats (Litter Mates) with $10^3$ W-256 Cells when Rats were 6 weeks Old*

| Radiation–inoculation interval (days) | Final body weight (g) | Number of lung colonies | Spleen weight (g) | Thymus weight (g) |
|---|---|---|---|---|
| 0 | 147 | 9±3 | 0·34 (0·219)* | 0·19 (0·122)* |
| 14 | 121 | 57±13 | 0·80 (0·640)* | 0·18 (0·144)* |

* Mean weights of spleen and thymus expressed per unit body weight ($\times 10^2$) shown in brackets.

TABLE III.—*Colony Counts ($N_L$) and Organ Weights Compared for Rats on Unrestricted Diet Inoculated at 42 Days of Age (Group A) with those in Younger Rats Inoculated at 35 Days (Group C) and at 28 Days (Group D) Respectively, and with Rats Inoculated at 42 Days, whose Weight had been Reduced by Restriction of Diet for 1 Week Preceding Inoculation (Group B). All Rats were given 570 rad WBI 24 hours Preceding Inoculation of 2 × 10² W-256 Cells and were Killed 7 Days Later. Ascites Tumour from a Single Donor Rat was used to Inoculate Rats in all 4 Groups on the Same Day. Column $W_I$ shows Ages and Body Weights at the Time of Inoculation, $W_S$ the Ages and Body Weights 7 Days later when Rats were Killed*

| Group (10 rats per group) | Age in days (Body weight Wg) | | | | Change in Mean Body Weight: 7 days preceding inoculation | Change in Mean Body Weight: 7 days after inoculation | $N_L$ | Mean Organ Weight g (per unit body weight $gg^{-1} \times 10^2$): Spleen | Thymus |
|---|---|---|---|---|---|---|---|---|---|
| | | | $W_1$ | $W_s$ | | | | | |
| A | 28 (71±0·5) | 35 (111±1) | 42 (131±2) | 49 (152±4) | $W_{42}-W_{35}$ = +20 g | $W_{49}-W_{42}$ = +21 g | 2·8± 0·7 | 0·24 (0·157) | 0·20 (0·131) |
| B (Tapioca diet from 35–42 days of age only) | 28 (71±0·5) | 35 (110±1) | 42 (86±1) | 49 (121±2) | $W_{42}-W_{35}$ = −24 g | $W_{49}-W_{42}$ = +35 g | 2·4± 0·7 | 0·24 | 0·20 |
| C | — | 28 (69±0·5) | 35 (109±1) | 42 (131±2) | $W_{35}-W_{28}$ = +40 g | $W_{42}-W_{35}$ = +22 g | 36±8 | 0·26 (0·198) | 0·22 (0·167) |
| D | — | — | 28 (70±0·5) | 35 (93±1) | — | $W_{35}-W_{28}$ = +23 g | 31±4 | 0·20 (0·215) | 0·14 (0·150) |

Diet restricted to tapioca and water (*ad libitum*) with added vitamins *for one week before tumour inoculation only* to reduce body weight. Groups A, C and D received balanced diet *ad libitum* throughout.

twenty-five-fold. This effect of ALS was accompanied by marked increases in weight of spleen and thymus of rats of all ages, in contrast to marked decreases in weight of these organs persisting at 7 days after WBI.

6. *Immunized recipients*

Lung and kidney colony production by Y-P388 cells injected intravenously as a secondary challenge in rats immunized with HR tumour cells were markedly reduced (Table IV). In a similar experiment in 40-day old immunized rats injected with 10⁴ Y-P388 cells, lung colony production ($N_L = 3 \pm 0{\cdot}7$) was not increased by sublethal WBI given immediately preceding the inoculation ($N_L = 2 \pm 0{\cdot}5$). In older rats immunized

TABLE IV.—*Effects of Immunization with Heavily Irradiated (HR) Y-P388 Cells on Tumour Colony Formation in Mature Female Rats (160–190 g Body Weight) Inoculated with 10³ or 10⁴ Y-P388 Cells Intravenously. Group A, Whole Body Irradiation (570 rad) 24 Hours before Inoculation; B, no Irradiation; C no Irradiation but given 5 × 10⁷ HR Cells Twice Weekly for 3 Weeks Preceding Inoculation. Six Rats per Group Killed 7 Days after Inoculation*

| Number of tumour cells inoculated intravenously | Number of lung colonies: Group A | Group B | Group C |
|---|---|---|---|
| 10³ | 16±4 (0·8; 0–3)* | 10±2 (0·2; 0–1) | 1·3±0·6 (0·2; 0–1) |
| 10⁴ | 141±39 (7·0; 1–20) | 17±5 (0·5; 0–2) | 0·3±0·2 (0·0) |

* Figures in brackets represent mean number (and ranges) of subcapsular tumour colonies in kidneys.

with HR tumour cells, or by growth of tumour in muscle (see Table I), a single dose of ALS was only slightly more effective than WBI in increasing the yield of lung colonies produced by an intravenous inoculation of the tumour (second challenge). When the number of ALS injections was increased to 3–5 doses before and after inoculation of the tumour, tumour colony production efficiencies rose to ~0·05 (Y-P388) and ~0·10 (W-256) in those rats surviving 7 days after inoculation.

7. *RES and age-dependent changes in tumour colony production*

Since WBI and ALS treatment are proven immunosuppressive treatments which modified clonogenic growth of these allogeneic tumours in lungs of rats, it seemed likely that some function(s) of the reticuloendothelial system (RES) might be implicated. Therefore, various supplementary experiments were performed using either 3–4 week old or older animals, and treating the rats before or after inoculation with steroids or cytotoxic chemicals or by splenectomy preceding inoculation. Treatment of animals with compound 48/80 to deplete tissue amines (Feldberg and Talesnik, 1953) has been found to increase growth of a heterologous tumour in rats (van den Brenk and Upfill, 1958) and to increase survival of skin homografts in rats (Boyd and Smith, 1960). Its effect on tumour colony growth was also studied. Most of these data are not given in full but are summarized to indicate whether or not any significant changes in colony forming efficiency were produced.

(a) *Steroids: Compound 48/80.*—Daily treatments with hydrocortisone (10 mg/kg) or dexamethasone (10 mg/kg) given intramuscularly, commenced 2 days before tumour inoculations and continued for 5 days afterwards, did not significantly alter the resistance of older rats to clonogenic growth of the tumours in lung or kidney. To deplete tissue biogenic amines compound 48/80 was injected intraperitoneally (daily) for 5 days. The first dose given was 100 μg and this was progressively increased by 100 μg per day, to a final dose of 500 μg on the fifth day, when rats were inoculated 4 hours later with $10^3$ W-256 cells. This treatment had no effect on CFE in either young or old rats.

(b) *Chemotherapeutic agents.*—Five doses of cyclophosphamide (20 mg/kg) or hydroxyurea (250 mg/kg) given intraperitoneally to rats over 7 days preceding tumour inoculation did not increase lung colony production significantly in older rats and were much less effective than WBI in weanlings.

(c) *Splenectomy.*—Splenectomy, 2–3 days before irradiation did not increase colony formation. When it was supplemented by WBI or local irradiation (1500 rad) to the thymus, splenectomy did not reverse age-dependent resistance to growth of Y-P388 or W-256 tumour cells in the lungs.

(d) *India ink.*—Quantitative studies of the granulopectic activity of the RES in rats have shown that loading of phagocytes with india ink reduces their capacity to remove further ink from the circulation, and produces a state of " saturation " which causes phagocytic function to deteriorate (Biozzi, Benacerraf and Halpern, 1953). Pretreatment of rats with a single large intravenous injection of 0·3 ml of india ink (Pelikan) given with a view to inhibiting the functions of macrophages present in the various tissues of the rat, had no significant effect on CFE, neither in weanling nor in older rats.

(e) *Whole body radiation dose.*—The whole body dose was increased to 900 rad in weanling rats and in older rats. Six-week old rats survived but a third of the weanlings had died within 7–10 days. Increasing the WBI dose did not increase tumour colony yields in older rats and fewer colonies were formed in weanling rats which survived than were seen after 570 rad. However, 900 rad caused early diarrhoea and rats lost weight very rapidly. This must be taken into account since inanition and severe loss of weight caused by irradiation or other treatments invariably inhibited the take and growth of these tumours in lungs and also their growth rates in muscle.

8. *Adoptive immunity*

In 3-week old rats given 900 rad WBI a large number ($1{\cdot}5 \times 10^7$) of bone marrow cells harvested from normal 8–10 week old donor rats, were injected intravenously 1–2 hours after $10^2$–$10^5$ Y-P388 tumour cells had been injected by the same route. The same treatments were given in parallel groups of rats using bone marrow cells harvested from donor rats previously immunized with HR tumour (Y-P388) cells. The results are shown in Fig. 6. Treatment with normal bone marrow cells caused $N_L$ to rise significantly in rats injected with $10^3$–$10^5$ cells and produced corresponding increases in lung weight but significantly reduced colonies formed by a small ($10^2$) cell inoculum. Bone marrow cells from immunized donors caused significant reductions in $N_L$ and $N_K$ after $10^2$–$10^5$ cells had been injected and corresponding reductions in lung weight. It is suggested that reductions in clonogenicity of tumour cells in lungs (and kidneys) by a large number of sensitized bone marrow cells is due to an adoptive immunity. Normal bone marrow cells are less efficient in this respect and are only effective in reducing growth of relatively fewer injected tumour cells, but give rise to a secondary " growth stimulating " effect which predominates when a larger number of tumour cells are present and apparently capable of exhausting the immune component (see Discussion).

Similar experiments were conducted with excesses of spleen cells, lymphocytes, thymocytes or peritoneal macrophages ($10^6$–$10^7$ cells) but these were *added* to each tumour cell inoculum. Spleen cells were obtained from 3–4 week old or 8–10 week old donors, or from 6-week old immunized donors. The recipients were weanling rats given 570 rad WBI. Normal spleen cells reduced tumour colonies produced in lungs and kidneys by $10^2$–$10^4$ Y-P388 cells by approximately 20%, donor spleen cells from young and old rats being equally effective; immunized spleen cells reduced colonies formed by $10^4$ tumour cells by approximately 50%. Similar experiments with lymphocytes, thymocytes and macrophages had no significant effects on colony production. These results will be reported later.

### DISCUSSION

Lung colonies produced by tumour cells injected intravenously have been described as " metastases ". Clearly, this term should not be used since the formation of metastases is a spontaneous event which depends primarily on the exfoliation of cells from a solid tumour and their capacity to enter lymph and blood vessels. However, arrest and growth in the lungs of tumour cells injected intravenously do provide a means for studying certain pathophysiological mechanisms involved in the formation of metastases. Colony forming efficiencies (CFE) reported for a variety of transplantable tumours (Table V) are comparatively low even if an immunosuppressive agent (WBI) had been used in syngeneic hosts to decrease their resistance due to possible antigenicity of tumour (Hill and Bush, 1969). Low CFE, taken in conjunction with their finding that CFE in syngeneic mice increased thirty-fold if an excess of radiation sterilized (HR) tumour cells or plastic microspheres were added to the viable cell inoculum, suggests that factors other than transplantation immunity may affect take and clonal growth of tumour cells present in the blood of an animal. Clarification of these factors would appear to be of considerable importance to the understanding of metastasis formation. Many clinical studies have demonstrated the presence of cancer cells in the blood of patients. Yet metastases may not develop

| SURVIVORS | N: $10^2$ | $10^3$ | $10^4$ | $10^5$ |
|---|---|---|---|---|
| ● *day 5* | 5 | 4 | 4 | 6 |
| ▲ *day 7* | 6 | 6 | 6 | 6 |
| ■ *day 7* | 6 | 6 | 6 | 6 |

FIG. 6.—Effect of treatment with bone marrow cells on tumour colony counts in lungs ($N_L$) and kidneys ($N_K$) of weanling rats 5 or 7 days after intravenous injection of Y-P388 cells. All rats received WBI immediately preceding inoculation of tumour and were killed 5 or 7 days after inoculation. Key to symbols: ———— 570 rad WBI (previous data, see Fig. 1)
- - - - ● - - - - 900 rad WBI
- - - - ▲ - - - - 900 rad WBI (1·5 × $10^7$ normal rat bone marrow cells injected intravenously 1 hour after tumour cells)
- - - - ■ - - - - 900 rad WBI (1·5 × $10^7$ sensitized rat bone marrow cells injected intravenously 1 hour after tumour cells).

On the fifth day after inoculation some rats from groups inoculated after 900 rad WBI but not treated with bone marrow, had died (see survival data in Fig. 6); the survivors were moribund and killed on this day to measure $N_L$ ($N_K$ could not be measured because presence of kidney colonies was masked by renal congestion). Rats in all other groups were killed on the seventh day to measure $N_L$ and $N_K$.

TABLE V.—*Tumour Colony Forming Efficiency (CFE Mean Number of Macrocolonies Produced in Lungs ($N_L$) per Tumour Cell Injected Intravenously) Reported in Literature. N number of Tumour Cells Injected; Exponent θ Calculated from the Data if* $N = kN_L\theta$ *(see Text)*

| Tumour | $N$ | Host | CFE | $\theta$ | Reference |
|---|---|---|---|---|---|
| Ehrlich tumour | 9–16 ($\times 10^5$)* | unirradiated 4–6 month old mice (allogeneic) | $<0\cdot0001$ | 3·28 | Baserga *et al.* (1960) |
| Polyoma virus transformed (f12) rat cells | 2–5 ($\pm 10^3$) | weanling rats (allogeneic) | | | |
| | | (a) irradiated | $\sim0\cdot015$ | 1·03 | Williams and Till (1966) |
| | | (b) unirradiated | $<0\cdot005$ | | |
| KHT—sarcoma | 1–15 ($\times 10^2$) | unirradiated 5–7 week old C3H mice (syngeneic) | $\sim0\cdot001$ ($\sim0\cdot03$ by adding HR cells) | 0·92 | Hill and Bush (1966) |
| Y-P388 sarcoma | $10^2$–$10^5$ | irradiated weanling rats (allogeneic) | $>0\cdot01$ | 0·72 | |
| W-256 carcinoma | 10–$10^3$ | irradiated weanling rats (allogeneic) | $>0\cdot1$ | 0·93 | |

* Values of $N$ used to calculate $\theta$ which produced one or more lung colonies in all mice injected; mice injected with $\sim 9 \times 10^4$ Ehrlich cells rarely developed visible lung colonies 30 days later.

under conditions in which evidence of autoimmunity to tumour growth is lacking and this suggests that the mechanisms which provide surveillance and defence against circulating tumour cells are not entirely attributable to classic immune reactions.

Hewitt (1953) showed that C3H suckling mice inoculated subcutaneously with very small numbers of allogeneic (S37) tumour cells were as highly susceptible to growth of this tumour as to growth of a syngeneic C3H sarcoma. However, susceptibility to S37 decreased rapidly with increase in age (particularly during the first week of life), whereas susceptibility of older mice to take and growth of the syngeneic tumour remained high. Similar experiments of our own with Y-P388 and W-256 tumours transplanted intramuscularly in rats have shown that ageing caused no significant decrease in susceptibility to growth of either tumour in muscle. $ED_{50}$ values after weaning were relatively constant, $\sim 5 \times 10^3$ Y-P388 cells and $<10$ W-256 cells respectively. On the other hand, take and clonogenic growth of cells of the tumours in lungs of rats based on macrocolony formation decreased relatively rapidly with increase in age after weaning, *even when rats had been exposed to sublethal WBI to suppress their immunity*—a treatment which reduced the $ED_{50}$ value for a primary challenge of Y-P388 tumour in muscle to $<10$ cells and greatly increased lethality and growth of metastases produced by this tumour in lymph nodes and lungs (van den Brenk *et al.*, 1971*a*). Resistance to growth of a primary challenge of Y-P388 or W-256 cells in the lungs caused by ageing of host, however, could be largely eliminated by heterologous anti-rat lymphocytic serum (ALS). In transplantation systems ALS is a powerful immunosuppressive agent (Woodruff, 1960), the most powerful yet described, but its exact mechanism of action remains uncertain (Levey and Medawar, 1966). Primarily its action in this respect has been variously attributed to lymphocytolysis, acting as a competitive antigen, coating and "blindfolding" lymphocytes so as to prevent recognition of antigen by lymphocyte, or to some other effects but the result of its actions *in vivo* is "to weaken the reactive capabilities of already sensitized animals to a degree that approaches a complete erasure of immunological memory" (Levey and Medawar, 1966). This effect of ALS of suppressing the secondary immune response to the growth of an allogeneic tumour transplanted in mice which had previously rejected a tumour transplant has been demonstrated by Riches and Thomas (1970), who also showed that sublethal WBI was ineffective in this respect and would only suppress immunity to a primary challenge of the tumour. In our own system older rats reacted to a primary intravenous challenge with tumour cells like " sensitized " animals since resistance to tumour growth, which increased spontaneously with increase in age of host, could be suppressed by ALS but not by WBI. On the other hand, the resistance of both weanling and older rats to a primary challenge of Y-P388 tumour in muscle was reduced by either WBI or ALS; irradiation did not significantly reduce the secondary response of the host to tumour growth in muscle whereas ALS did so but not completely (unpublished results). The finding reported by Fisher, Soliman and Fisher (1969) that ALS enhanced take, growth and metastasis of mouse mammary tumours in syngeneic C3HB/FeJ hosts is also of interest in this respect. Certain other findings also appear relevant to the mode of action of immunosuppressive agents against growth of syngeneic and allogeneic tumours. Thus Patterson *et al.*, (1970) consider that their experiments point to the macrophage being the primary cellular site of ALS induced immunosuppression. Other workers have provided evidence that ALS destroys or neutralizes the functions of a variety of circulating and fixed tissue cells other than lymphocytes, including other leucocytes, splenic giant cells and even epithelial and connective tissue elements (see Yoffey and Courtice, 1970). The glomerular mesangeitis produced rapidly by the nephrotoxic action of ALS on the kidney (Lindquist *et al.*, 1969) may be a further example of the nonspecific action of ALS on tissues.

The suppressive effects of WBI of host on the primary response to an allograft can be attributed largely to the destruction of bone marrow which prevents the elaboration of immunocompetent cells. This action should prevail at any age and decrease the primary immune response to macrocolony growth of antigenic tumour cells in the lungs, but our experiments have clearly shown that sublethal WBI did not decrease materially the resistance of older rats to macrocolony growth of tumour. Furthermore, when the injection of tumour cells was delayed for some days after WBI, CFE rose and at a time when regeneration of bone marrow and other immunocytic tissues (spleen, thymus) was progressing and the blood leucocyte count had been restored to near-normal. Also significant has been our finding that *local* irradiation of the lungs could be as effective as WBI in increasing tumour CFE in lungs of weanling rats and given under appropriation conditions *completely reversed the resistance of older rats to growth of a primary inoculum in the lungs* (to be published).

In attempting to explain our results, it seems possible that these two tumours possess certain histoincompatibility antigens to which weanling rats react weakly and that this reaction develops rapidly after weaning. Thus, immunoelectrophoretic studies of the antigenic profile of Yoshida sarcoma by Caputo (1969) showed the presence of several nonspecific components in addition to " $Y_1$ " and " $Y_3$ " organ specific antigens and a " $Y_2$ " tumour specific antigen occurring in both microsomal and mitochondrial cell fractions. But such " maturation " of immunological competence does not appear to explain completely continued susceptibility of older rats to growth of the tumour in muscle although it appears reasonable to assume that single cells which arrest " physiologically " in the lung, would be more vulnerable to any immune attack the host can offer than a " consortium " of the cells in muscle where trauma produced by inoculation may help to shield the inoculum from humoral defences. This argument can also be advanced to account for the breakdown of resistance to clonogenic growth of cells in *locally* irradiated tissues where inflammatory reactions conceivably *compete* for " immunocytes ", or the products of inflammation cause target cells to become less accessible to immunocytic destruction, or possibly interfere with immunochemical mechanisms (*e.g.* availability of complement).

A further possibility which we feel should not be excluded is that " growth stimulating substances " (GSS) of a non-specific nature are conducive to growth and survival of cells (*in vitro* and *in vivo*) and that tissue maturation with ageing may bring about low local concentrations of GSS which causes single tumour cells to grow poorly—a situation not based on immunity. The role of GSS in stimulating proliferative cell growth has been demonstrated in a variety of biological situations, *in vitro* and *in vivo*, including the continued production of GSS by heavily irradiated (HR) cells which stimulates tumour growth *in vitro* (Puck and Marcus, 1956) and *in vivo* (Revesz, 1958; van den Brenk and Sharpington, 1971*b*), a "leucocyte stimulating factor" in granulopoiesis (Metcalf, 1970) which is also present in serum of irradiated mice (Morley *et al.*, 1971; Rickard *et al.*, 1971*a*, *b*), a portal blood factor acting as a humoral agent in liver regeneration (Fisher *et al.*, 1971), a HeLa cell stimulating factor in normal calf serum (Salmon and Hosse, 1971) and polyamines derived from ornithine in tissues which stimulate normal and neoplastic growth (Tabor and Tabor, 1964; Bucher and Malt, 1971). Conditioning of the cellular environment by GSS produced by host tissues *in situ* may be a positive factor which " competes " with any immune reaction of the body to " take " and growth of tumour cells, and facilitates the ease with which grafted cells survive and begin to proliferate. Once replication of the sequestrated (tumour) cell is under way, a colony arises which itself produces GSS for growth to continue. Increase in population density of the colony would not only decrease the accessibility of its innermost cells to the destructive actions of immunocytes and their products, but, *vis a tergo* would stimulate growth in the colony by generating GSS *in situ*. It seems likely that ALS, like certain other heterologous sera and proteins (*e.g.* phytohaemagglutin), has blastogenic and growth stimulating actions *in vivo*, besides acting as an immunosuppressant. Conceivably, growth stimulating effects may help to promote tumour growth in allogeneic and syngeneic hosts. Reactions to tissue damage caused by irradiation lead to hyperplastic (regenerative) changes which also possibly cause GSS levels to rise. The finding that delaying inoculation after WBI of older rats increased tumour CFE in lungs would be in accord with this concept of conditioning by GSS and recent experiments (to be published) have shown that stimulation of growth after local lung irradiation also depends on the delay between irradiation and inoculation; by appropriately adjusting the radiation dose and the radiation–inoculation interval, resistance of older rats to tumour macrocolony growth in lung could be inhibited. Colony regression seen to occur in older rats is also considered to arise from stimulation of growth in the first instance, followed by suppression later when immunosuppressive forces dominate. A population of actively growing tumour cells provides the most effective antigeneic stimulus to the host (Haddow and Alexander, 1964), and this stimulus would develop during the growth of colonies. Low efficiencies of macrocolony production could thus be attributed to presence of microcolonies or to regression of colonies as well as to failed " takes " from lack of support and stimulation of cell growth. It seems significant that older recipient animals were used by Hill and Bush (1969), who also found that HR cells (a source of antigen and potentially capable of enhancing immunity) greatly increased CFE.

Prehn (1972) has recently provided further data in support of his theory that the effect of immunity on a target tumour cell is biphasic and consists of both a *mild* reaction which *stimulates* tumour growth and a strong cytotoxic reaction. The nature of this stimulatory component is not known but normal or " sensitized " syngeneic spleen cells brought into contact with the tumour cells *in vitro* stimulated tumour growth, at a critical spleen cell/tumour cell ratio of approximately $10^4 : 1$, higher spleen/tumour cell ratios inhibiting growth. These findings seem similar in nature to the effect of treating rats injected with W-256 cells with an excess of normal bone marrow cells (Fig. 7). A cytotoxic action against fewer tumour cells predominated, whereas take and growth of a larger number of inoculated tumour cells were stimulated by normal bone marrow but not by sensitized bone marrow.

In conclusion, it is clear that the use of a non-antigenic or weakly antigenic tumour in syngeneic or immunosuppressed hosts for macrocolony assays does not necessarily ensure high CFE. CFE is not affected significantly by sex of host, but mean lung colony numbers obtained in assays show large variances and individual lung colony counts do not conform to a Poisson distribution. The reasons for this variation are obscure but are not due simply to differences in immunological compatibility. The age of animals used in assays greatly affects CFE. This effect could not be reconciled with increases in body weight and lung volume with increase in age of rat. Animal age needs to be carefully controlled if results of cell assays are to have quantitative significance and other suitable measures which raise CFE (including immunosuppressant therapy) must be taken. Various attempts were made to improve CFE. Splenectomy, steroid therapy, depletion of tissue amines by compound 48/80, saturation of the granulopectic functions of the RES by india ink and treatment with cytotoxic drugs were ineffective; only WBI or ALS raised CFE. The rise in CFE produced by these treatments is considered due not only to immunosuppressant actions but also to a local " conditioning " by the agent of the tumour cell bed, which allows " take " and early replication of tumour cells to occur more readily.

### REFERENCES

BASERGA, R., PUTONG, P. B., TYLER, S. & WARTMAN, W. B. (1960) The Dose–Response Relationship between the Number of Embolic Tumour Cells and the Incidence of Blood-borne Metastases. *Br. J. Cancer*, **14**, 173.

BIOZZI, G., BENACERRAF, B. & HALPERN, B. N. (1953) Quantitative Study of the Granulopectic Activity of the Reticuloendothelial System. II. A Study of the Kinetics of the Granulopectic Activity of the RES in Relation to the Dose of Carbon Injected. Relationship between the Weight of the Organs and their Activity. *Br. J. exp. Path.*, **34**, 441.

BOYD, J. F. & SMITH, A. N. (1960) The Effect of Compound 48/80 on the Autograft and Homograft Reaction. *Br. J. exp. Path.*, **41**, 259.

BUCHER, N. L. R. & MALT, R. A. (1971) *Regeneration of Liver and Kidney.* Boston: Little, Brown & Co.

CAPUTO, A. (1969) Antigens of Yoshida Tumour Cells. *J. Path.*, **97**, 639.

FELDBERG, W. & TALESNIK, J. (1953) Reduction of Tissue Histamine by Compound 48/80. *J. Physiol.*, **120**, 550.

FISHER, B., SOLIMAN, D. & FISHER, E. R. (1969) Effect of Antilymphocyte Serum on Parameters of Tumour Growth in a Syngeneic Tumour—Host System. *Proc. Soc. exp. Biol. Med.*, **130**, 16.

FISHER, B., SZUCH, P., LEVEUE, M. & FISHER, E. R. (1971) A Portal Blood Factor as the Humoral Agent in Liver Regeneration. *Science, N.Y.*, **171**, 575.

GUTTMANN, R. D., CARPENTER, C. B., LINDQUIST, R. R. & MERRILL, J. P. (1967) Treatment with Heterologous Antithymus Sera: Nephritis Associated with Modification of Renal Allograft Rejection and the Immune Status of the Host to the Foreign protein. *Transplantation*, **5**, 1115.

HADDOW, A. & ALEXANDER, P. (1964) An Immunological Method of Increasing the Sensitivity of Primary Sarcomas to Local Irradiation with X-ray. *Lancet*, i, 452.

HEWITT, H. B. (1953) The Effect of Age of Host on the Quantitative Transplantation of Sarcoma 37. *Br. J. Cancer*, **7**, 384.

HILL, R. P. & BUSH, R. S. (1969) A Lung-colony Assay to Determine Radiosensitivity of the Cells of a Solid Tumour. *Int. J. Radiat. Biol.*, **15**, 435.

HUXLEY, J. S. (1932) *Problems of Relative Growth.* New York: Dial Press.

LEVEY, R. H. & MEDAWAR, P. B. (1966) Some Experiments on the Action of Antilymphoid Antisera. *Ann. N.Y. Acad. Sci.*, **129**, 164.

LINDQUIST, R. R., GUTTMANN, R. D., CARPENTER, C. B. & MERRILL, J. P. (1969) Nephritis Induced by Antilymphocyte Serum. *Transplantation*, **8**, 545.

METCALF, D. (1970) Studies of Colony Formation *in vitro* by Mouse Bone Marrow Cells. II. Action of Colony Stimulating Factor. *J. cell. comp. Physiol.*, **76**, 89.

MORLEY, A., RICKARD, K. A., HOWARD, D. & STOHLMAN, F. (1971) Studies on the Regulation of Granulopoiesis IV. Possible Humoral Regulation. *Blood*, **37**, 14.

PATTERSON, J. T., PISANO, J. C. & DI LUZIO, N. R. (1970) Reversal of Antilymphocytic Serum-induced Immunosuppression by Macrophage Administration. *Proc. Soc. exp. Biol. Med.*, **135**, 831.

PREHN, R. T. (1972) The Immune Reaction as a Stimulation of Tumour Growth. *Science, N.Y.*, **176**, 170.

PUCK, T. T. & MARCUS, P. I. (1956) Action of X-rays on Mammalian Cells. *J. exp. Med.*, **103**, 653.

REVESZ, L. (1958) Effect of Lethally Damaged Tumour Cells upon the Development of Admixed Viable Cells. *J. natn. Cancer Inst.*, **20**, 1157.

RICHES, A. C. & THOMAS, D. B. (1970) The Growth of Allogeneic Tumour Implants as an Index of Immunosuppression. *J. Anat.*, **107**, 392.

RICKARD, K. A., MORLEY, A., HOWARD, D., GARRITY, M. & STOHLMAN, F. (1971*a*) Stem Cell Stimulatory Properties *in vitro* of an Agar Colony-stimulating Factor. *Proc. Soc. exp. Biol. Med.*, **136**, 608.

RICKARD, K. A., MORLEY, A., HOWARD, D. & STOHLMAN, F. (1971*b*) The *in vitro* Colony-forming Cell and the Response to Neutropenia. *Blood*, **37**, 6.

SALMON, W. D. & HOSSE, B. R. (1971) Stimulation of HeLa Cell Growth by a Serum Fraction with Sulfation Factor Activity. *Proc. Soc. exp. Biol. Med.*, **136**, 805.

TABOR, H. & TABOR, C. W. (1964) Spermidine, Spermine and Related Amines. *Pharmac. Rev.*, **16**, 245.

VAN DEN BRENK, H. A. S., MOORE, V. & SHARPINGTON, C. (1971*a*) Growth of Metastases from P-388 Sarcoma in the Rat following Whole Body Irradiation. *Br. J. Cancer*, **25**, 186.

VAN DEN BRENK, H. A. S., SPARROW, N. & MOORE, V. (1969) Effect of X-radiation on Salivary growth in the Rat. 1. Effect of Single Doses on Post-natal Differentiation and Growth of Acinar and Duct Components. *Int. J. Radiat. Biol.*, **16**, 241.

VAN DEN BRENK, H. A. S. & SHARPINGTON, C. (1971*b*) Effect of Local X-irradiation of a Primary Sarcoma in the Rat on Dissemination and Growth of Metastases: Dose-Response Characteristics. *Br. J. Cancer*, **25**, 812.

VAN DEN BRENK, H. A. S. & UPFILL (1958) Heterologous Growth of Ehrlich Ascites Tumour in Histamine-depleted Rats. *Aust. J. Sci.*, **21**, 20.

WILLIAMS, J. F. & TILL, J. E. (1966) Formation of Lung Colonies by Polyoma-transformed Rat Embryo Cells. *J. natn. Cancer Inst.*, **37**, 177.

WOODRUFF, M. F. A. (1960) *The Transplantation of Tissues and Organs.* Springfield, Ill.: Charles C. Thomas.

YOFFEY, J. M. & COURTICE, F. C. (1970) *Lymphatics, Lymph and the Lymphomyeloid Complex.* London: Academic Press.

ZEIDMAN, I., McCUTCHEON, M. & COMAN, D. R. (1950) Factors Affecting the Number of Tumour Metastases. Experiment with a Transplantable Mouse Tumour. *Cancer Res.*, **10**, 357.


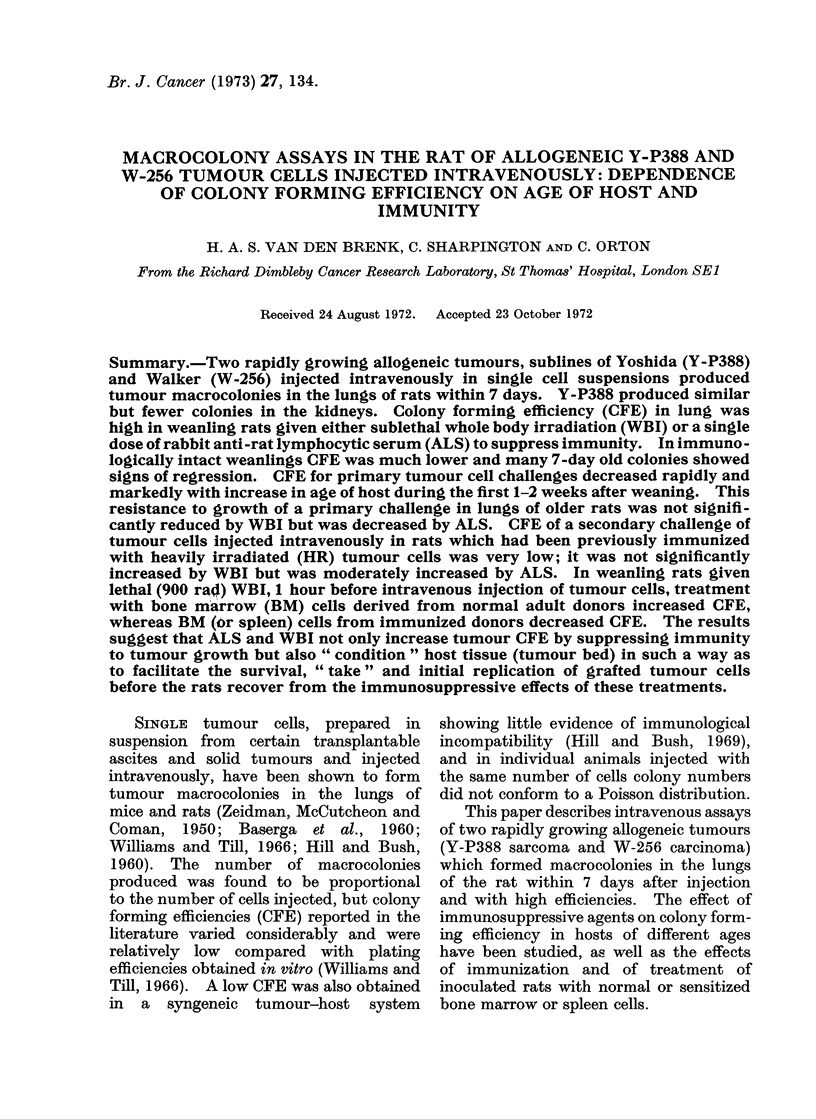

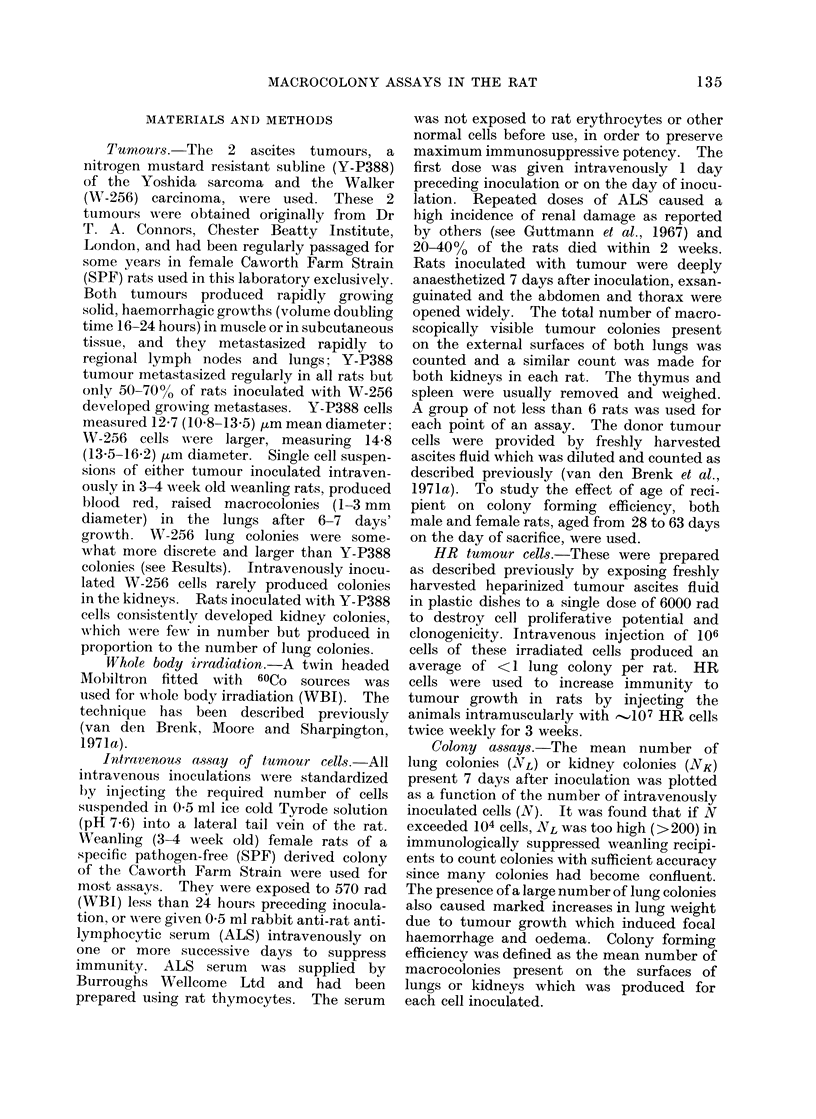

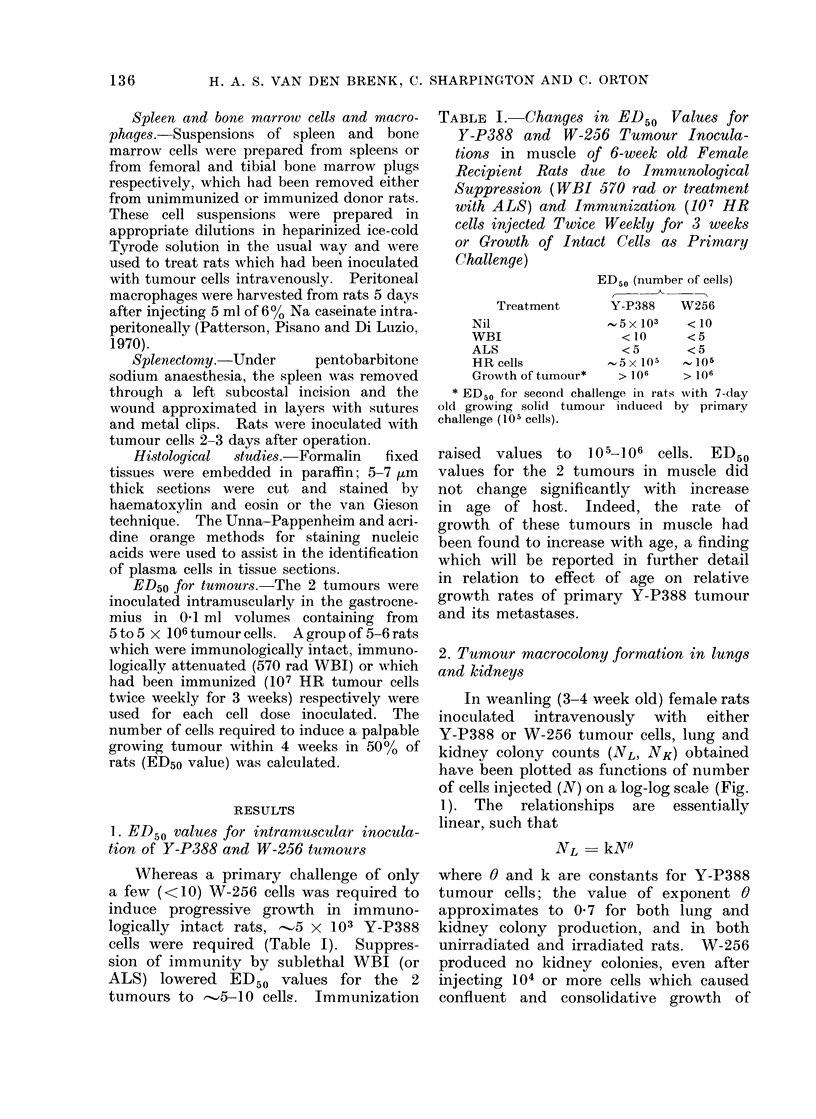

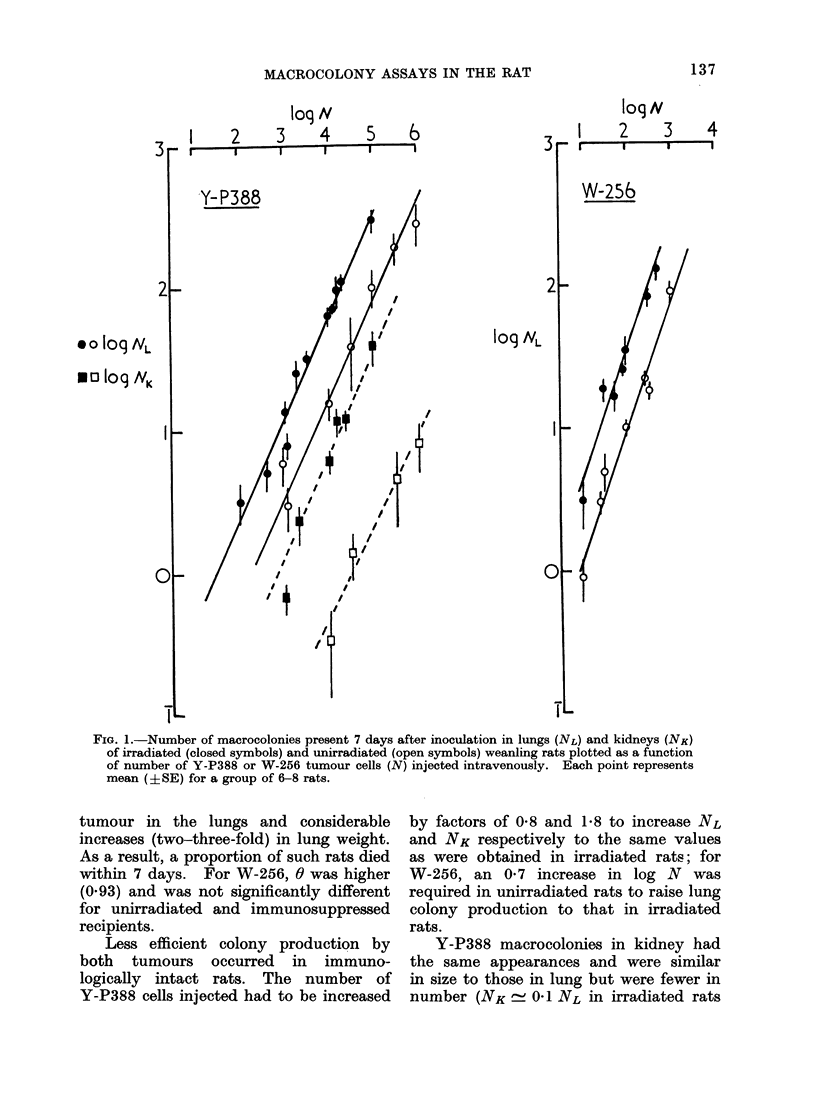

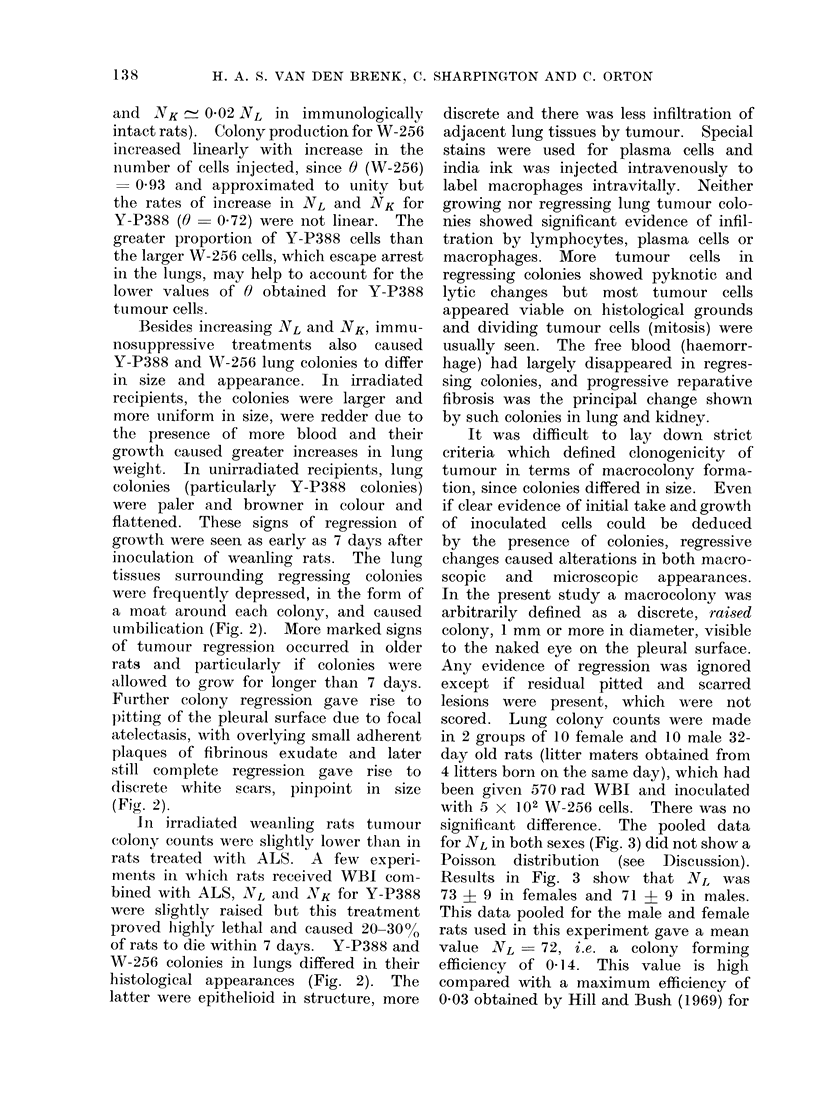

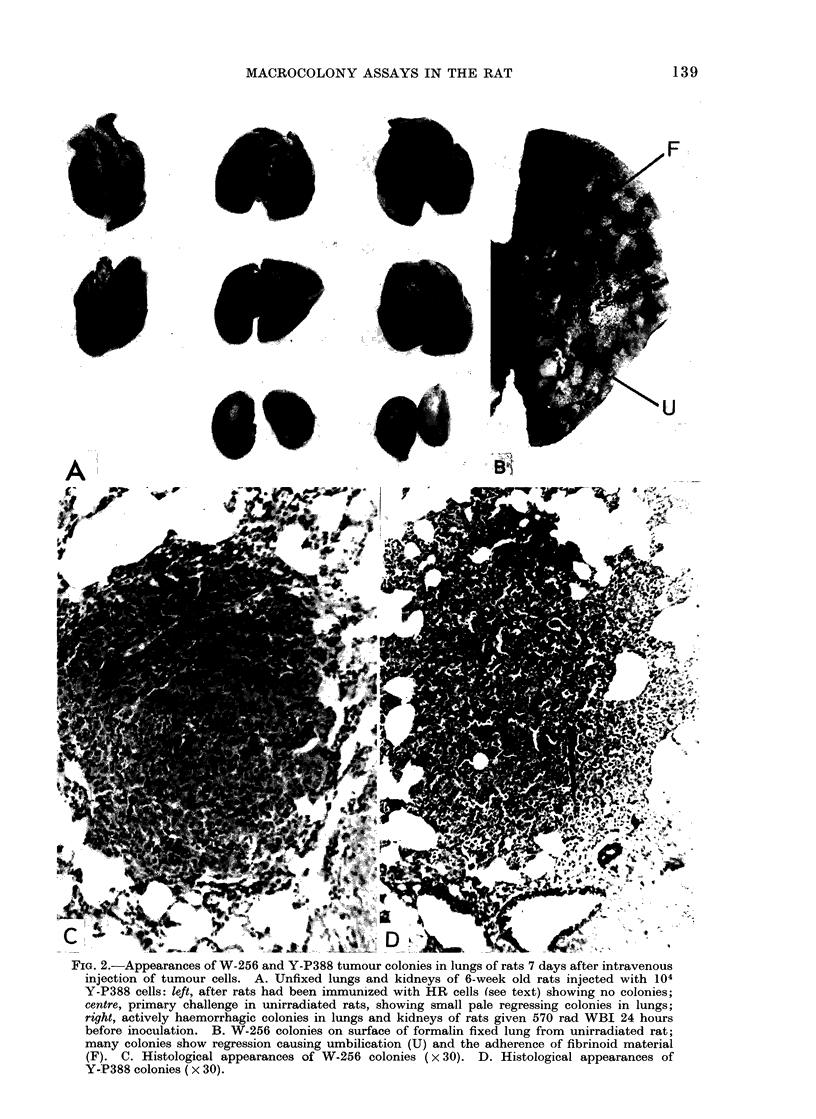

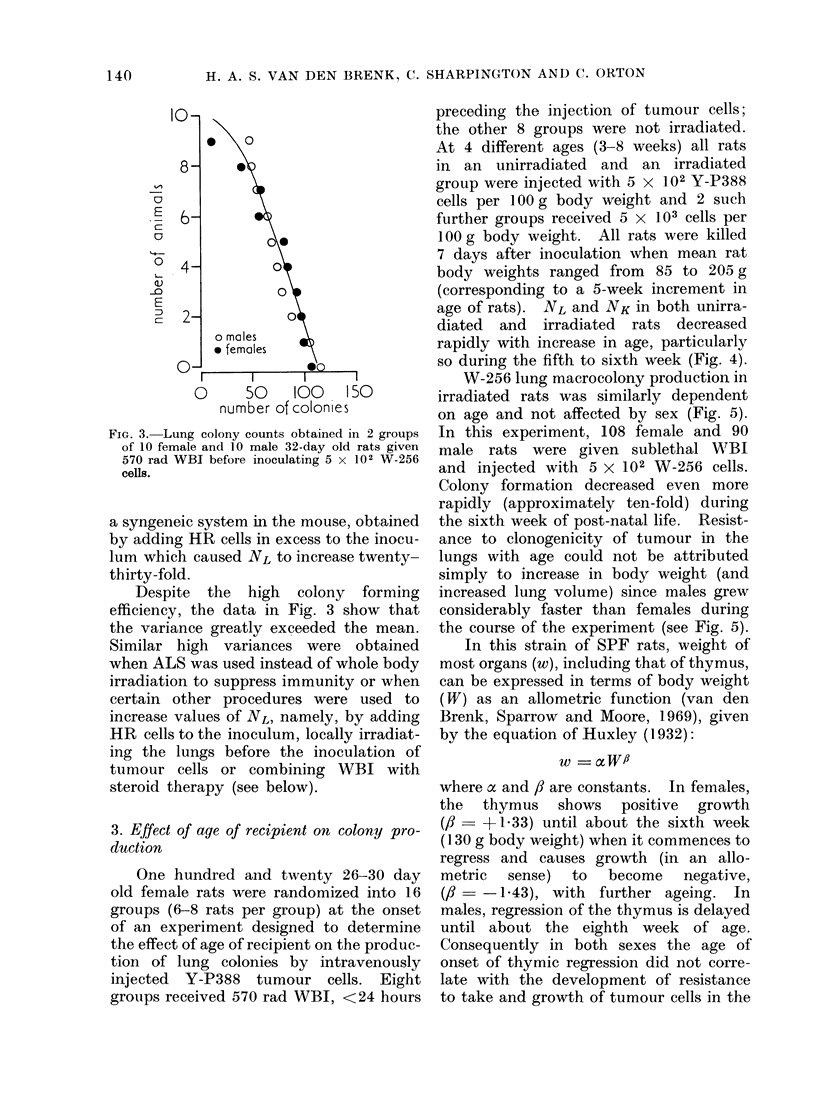

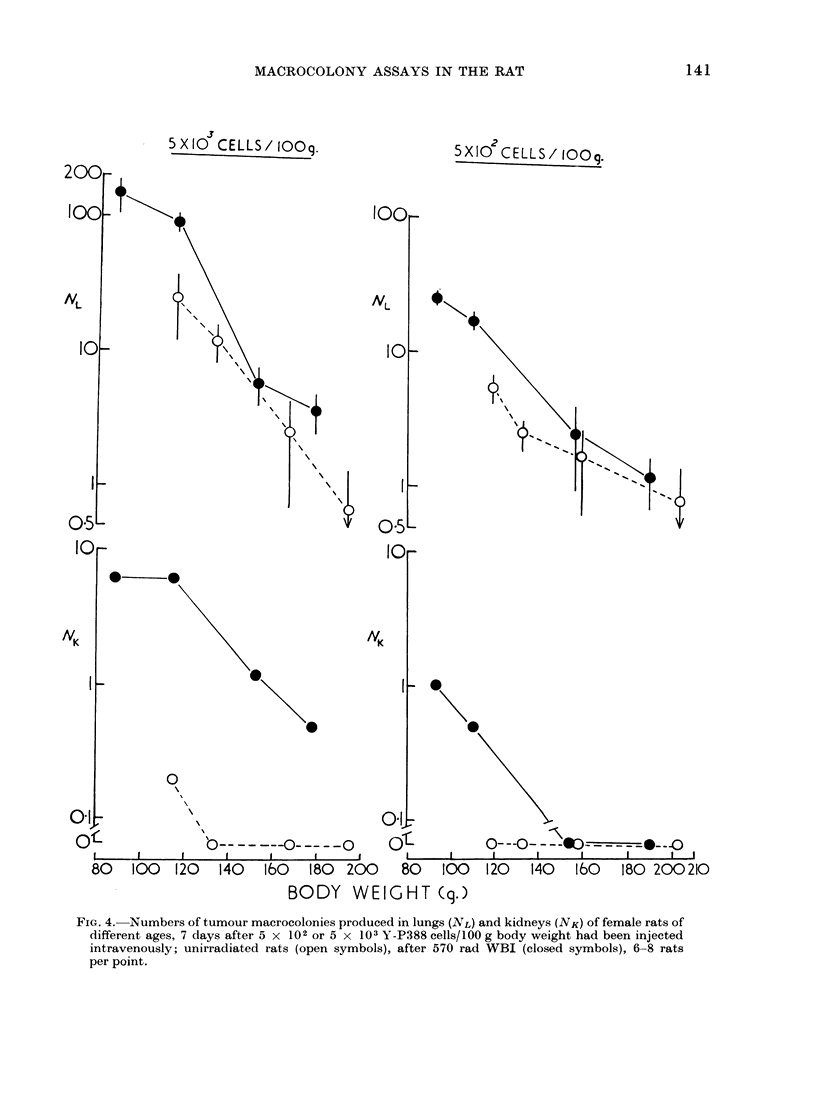

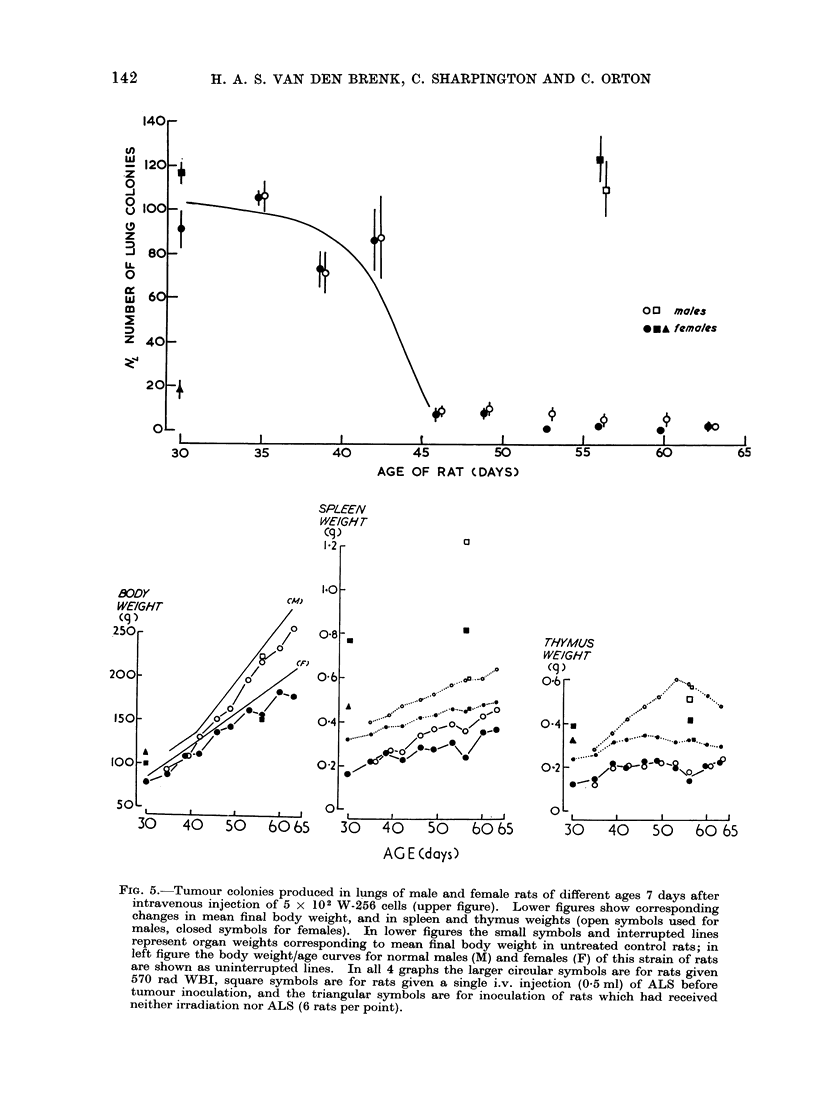

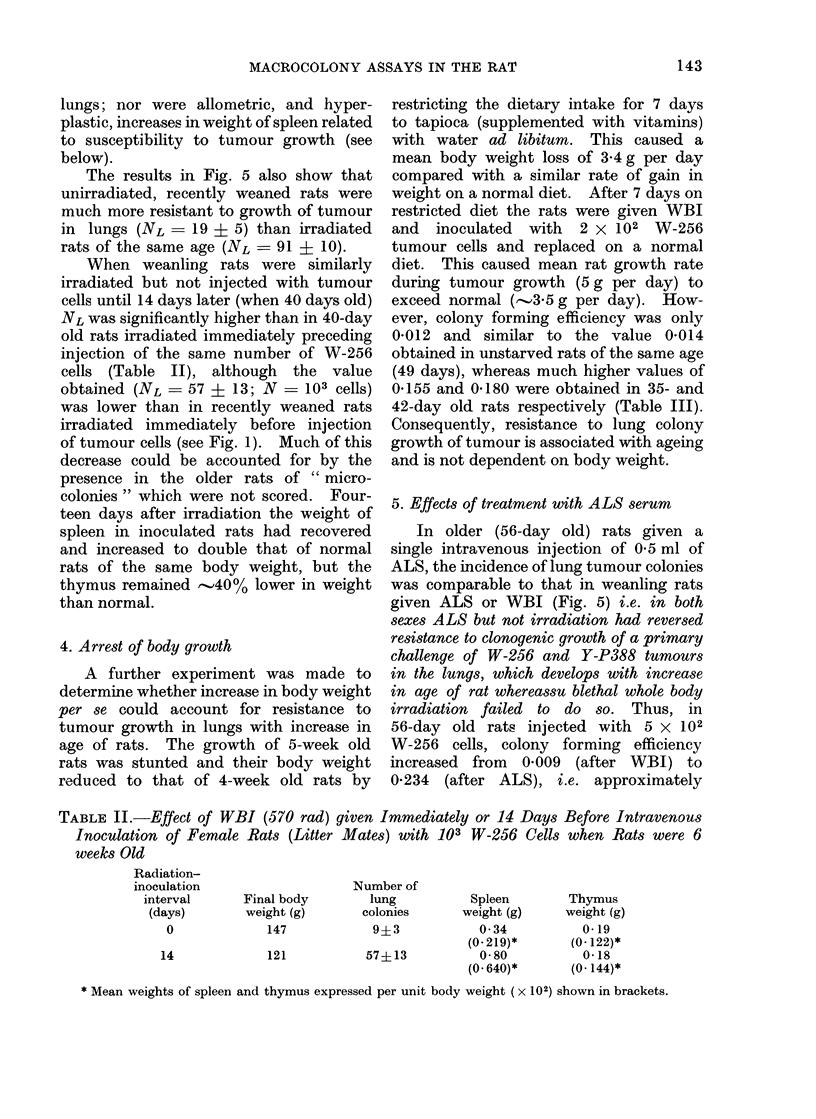

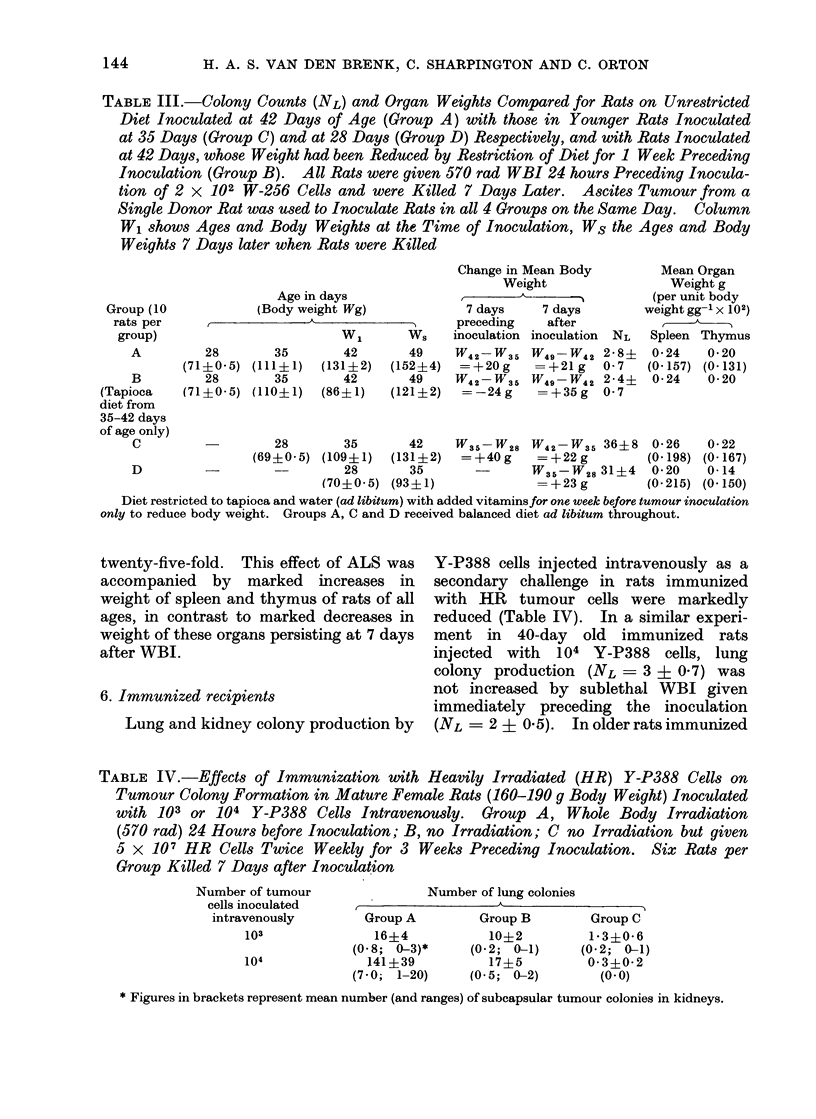

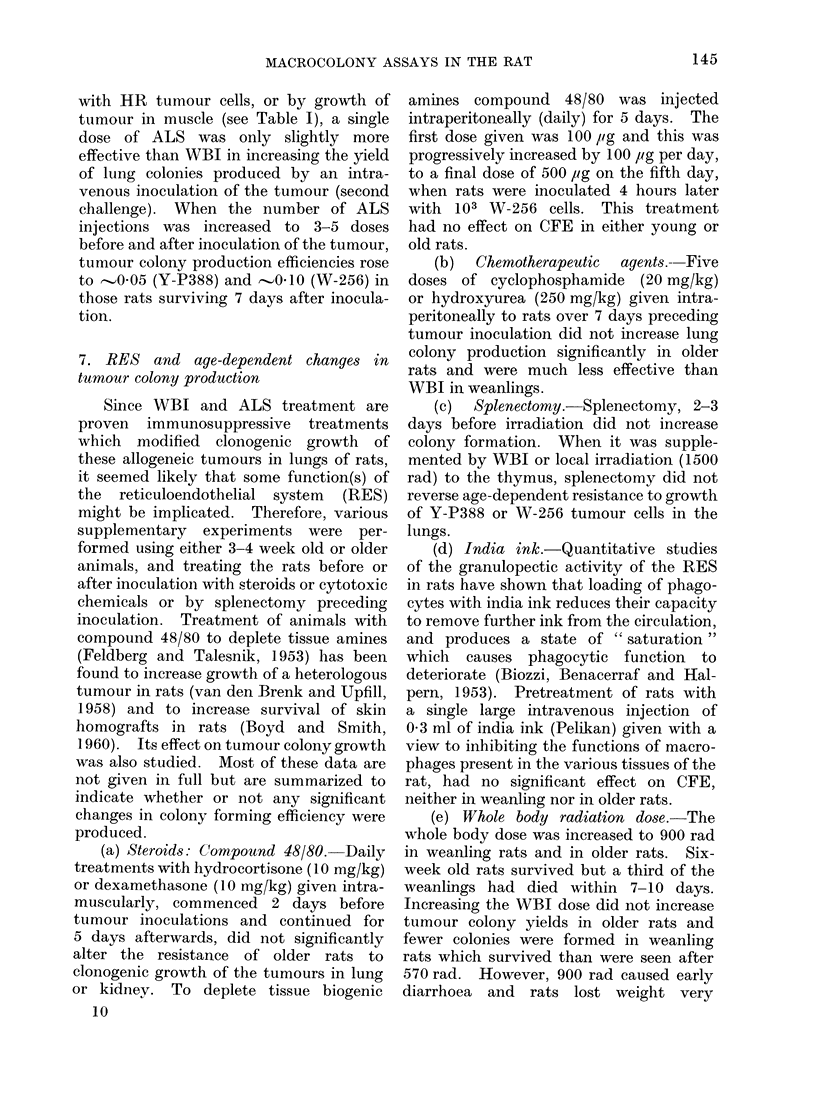

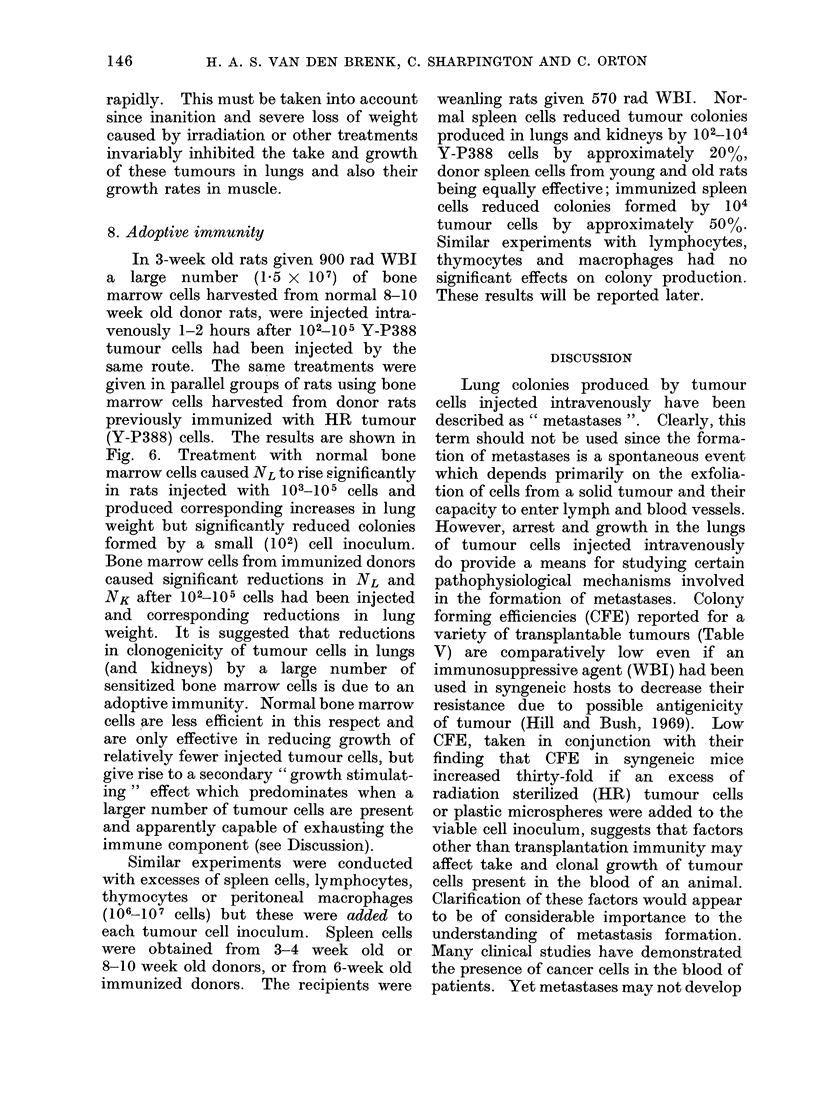

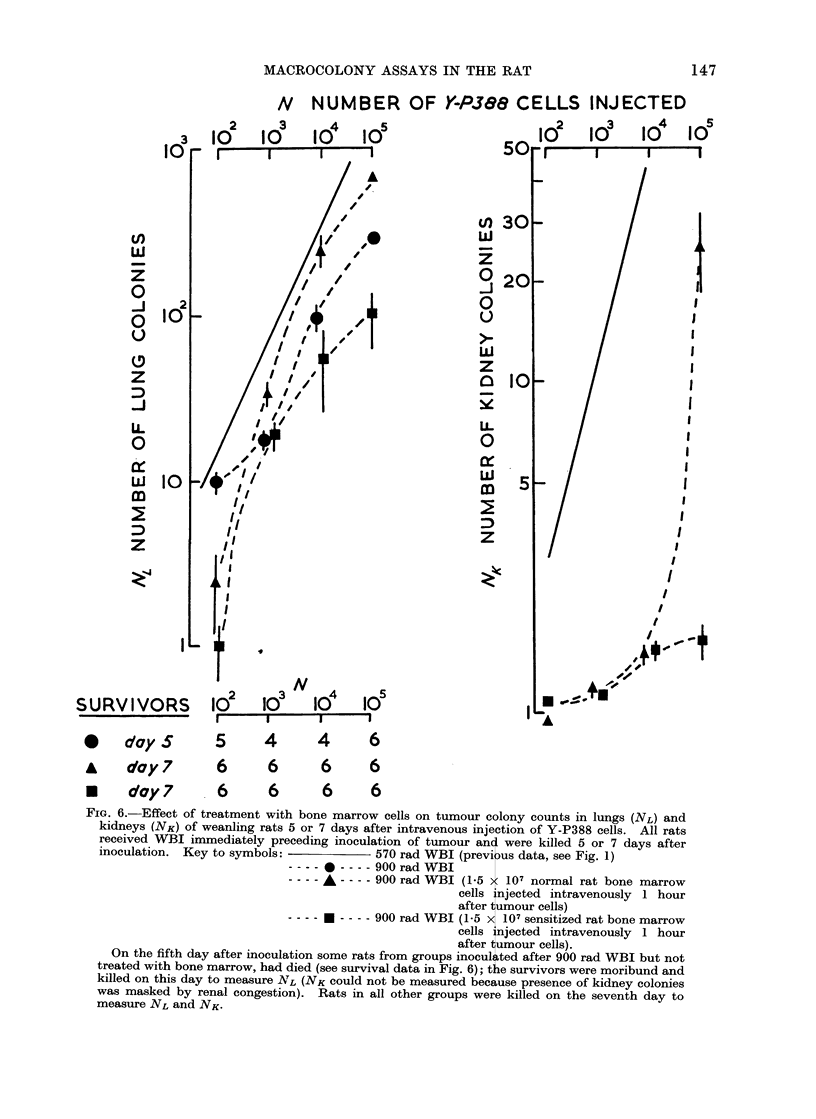

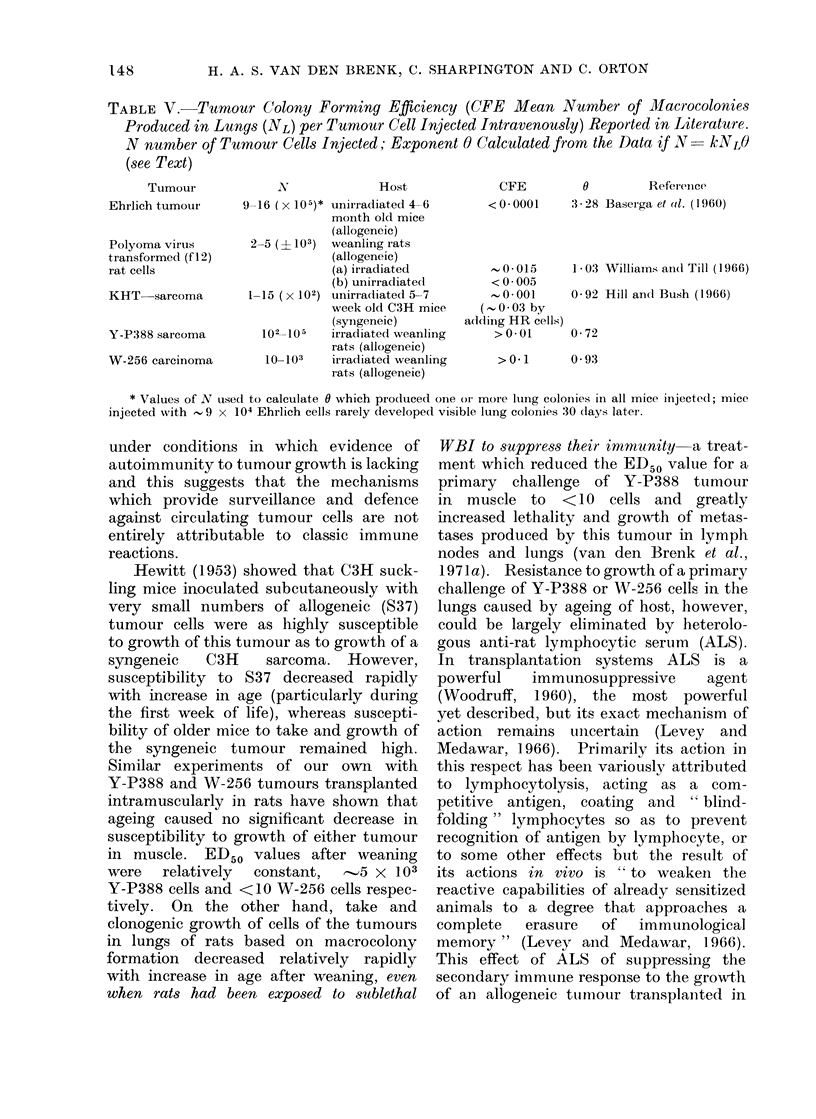

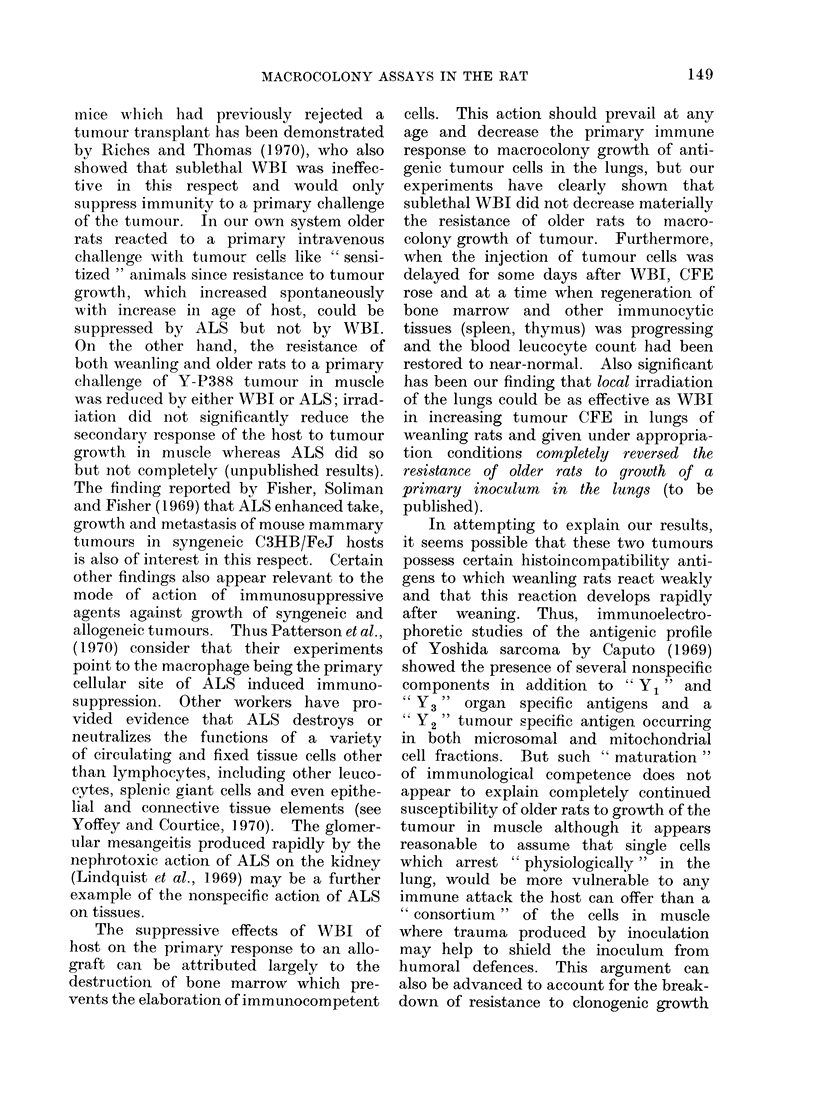

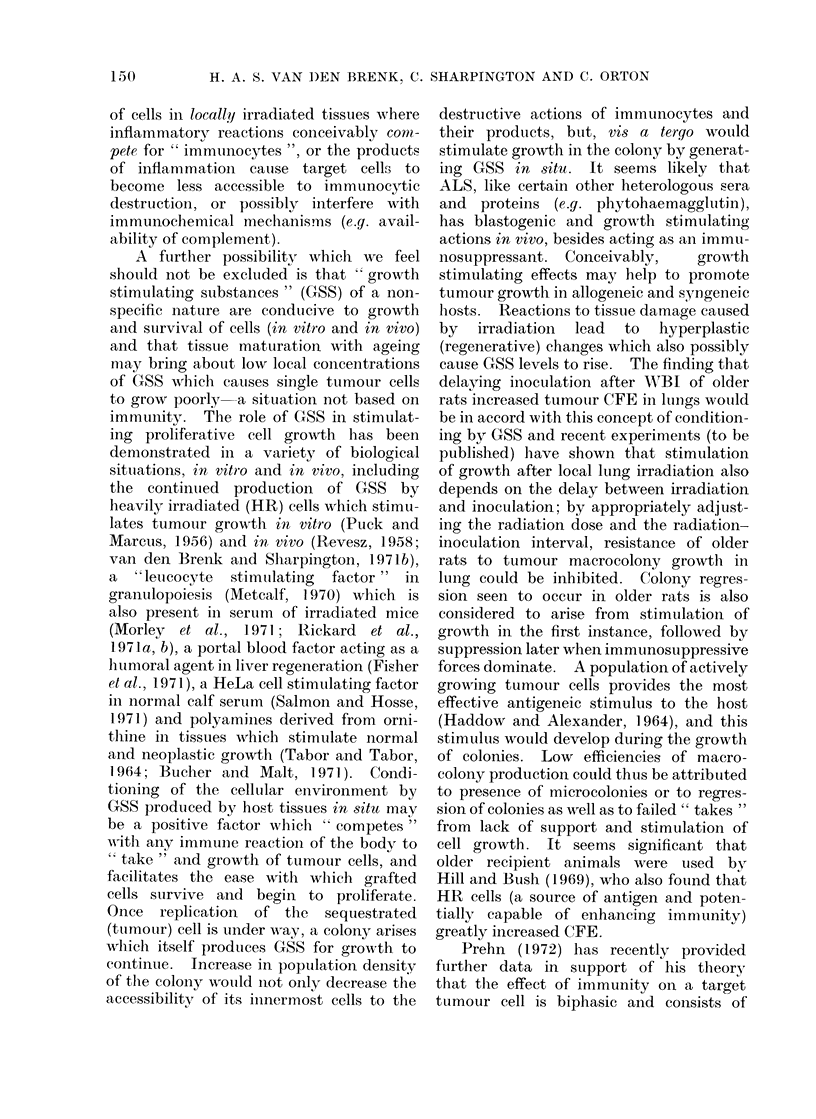

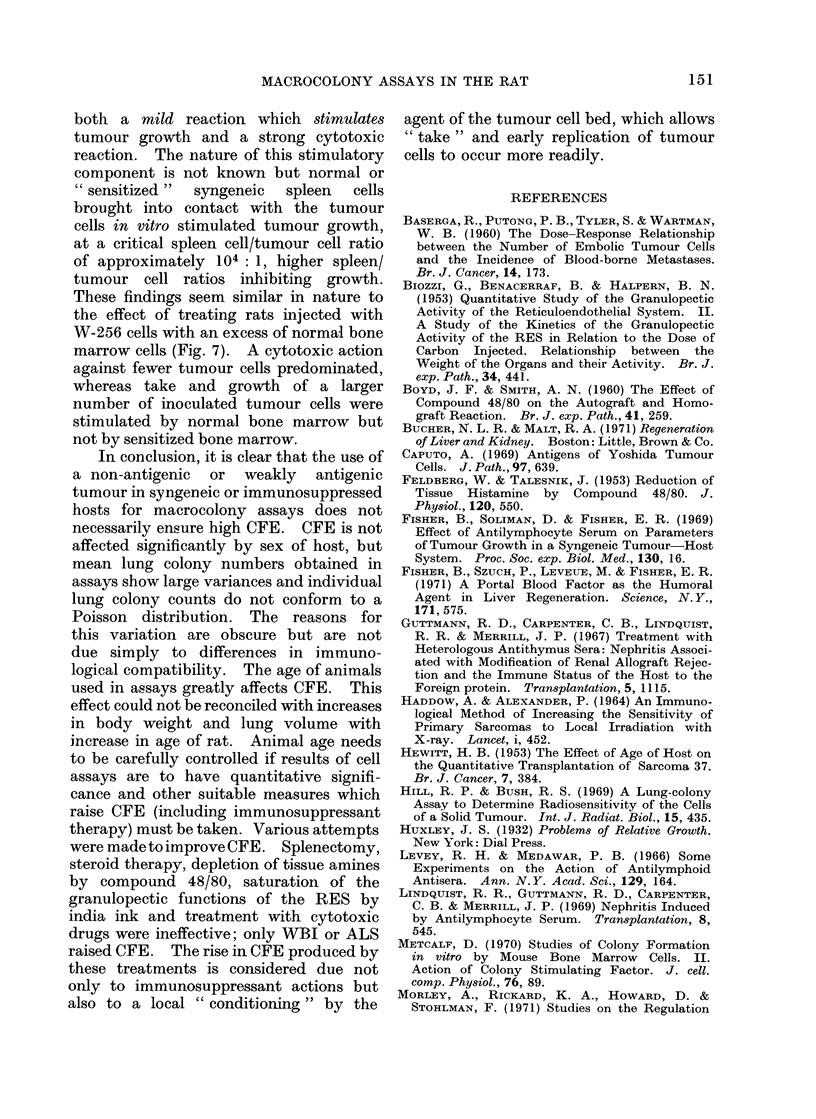

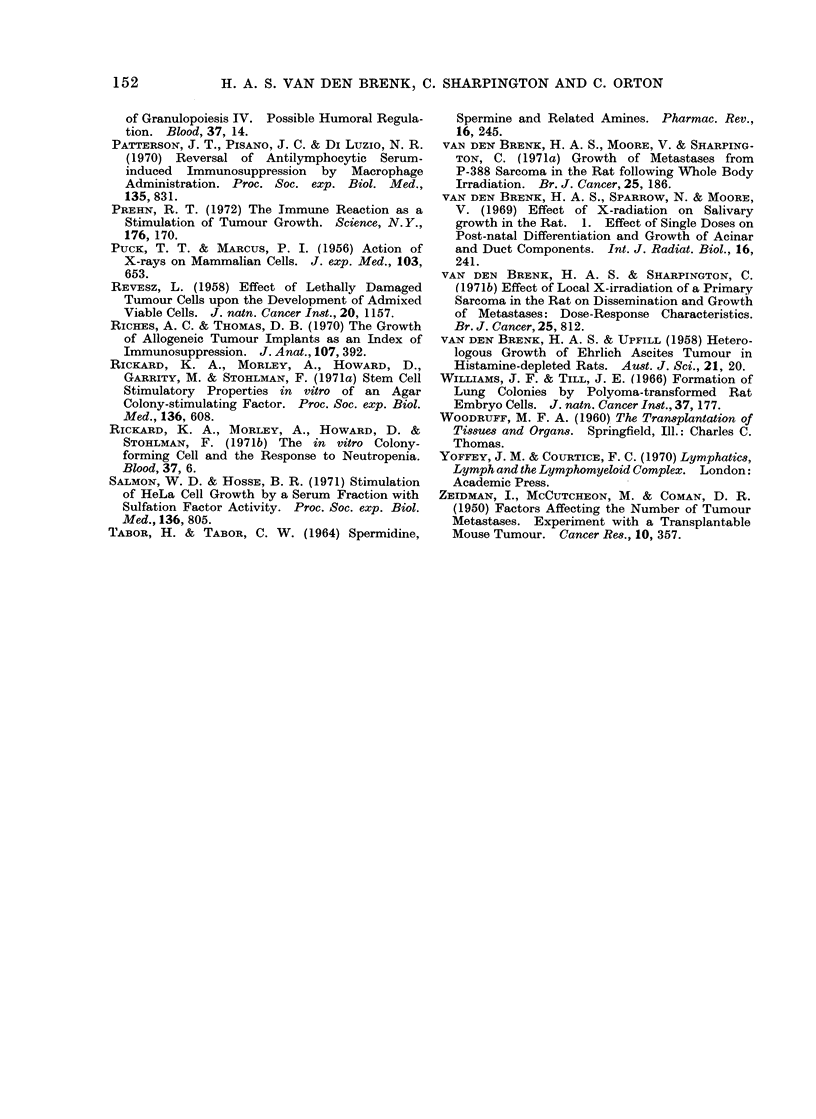


## References

[OCR_02043] BASERGA R., PUTONG P. B., TYLER S., WARTMAN W. B. (1960). The dose-response relationship between the number of embolic tumor cells and the incidence of blood-borne metastases.. Br J Cancer.

[OCR_02050] BIOZZI G., BENACERRAF B., HALPERN B. N. (1953). Quantitative study of the granulopectic activity of the reticulo-endothelial system. II. A study of the kinetics of the R. E. S. in relation to the dose of carbon injected; relationship between the weight of the organs and their activity.. Br J Exp Pathol.

[OCR_02060] BOYD J. F., SMITH A. N. (1960). The effect of compound 48/80 on the autograft and homograft reaction.. Br J Exp Pathol.

[OCR_02068] Caputo A. (1969). Antigens of Yoshida tumour cells.. J Pathol.

[OCR_02072] FELDBERG W., TALESNIK J. (1953). Reduction of tissue histamine by compound 48/80.. J Physiol.

[OCR_02077] Fisher B., Soliman O., Fisher E. R. (1969). Effect of antilymphocyte serum on parameters of tumor growth in a syngeneic tumor--host system.. Proc Soc Exp Biol Med.

[OCR_02089] Guttmann R. D., Carpenter C. B., Lindquist R. R., Merrill J. P. (1967). Treatment with heterologous anithymus sera: nephritis associated with modification of renal allograft rejection and the immune status of the host to the foreign protein.. Transplantation.

[OCR_02097] HADDOW A., ALEXANDER P. (1964). AN IMMUNOLOGICAL METHOD OF INCREASING THE SENSITIVITY OF PRIMARY SARCOMAS TO LOCAL IRRADIATION WITH X RAYS.. Lancet.

[OCR_02103] HEWITT H. B. (1953). The effect of age of host on the quantitative transplantation of sarcoma 37.. Br J Cancer.

[OCR_02108] Hill R. P., Bush R. S. (1969). A lung-colony assay to determine the radiosensitivity of cells of a solid tumour.. Int J Radiat Biol Relat Stud Phys Chem Med.

[OCR_02121] Lindquist R. R., Guttmann R. D., Carpenter C. B., Merrill J. P. (1969). Nephritis induced by antilymphocyte serum. An electron microscopic and immunohistochemical study.. Transplantation.

[OCR_02127] Metcalf D. (1970). Studies on colony formation in vitro by mouse bone marrow cells. II. Action of colony stimulating factor.. J Cell Physiol.

[OCR_02133] Morley A., Rickard K., Howard D., Stohlman F. (1971). Studies on the regulation of granulopoiesis. IV. Possible humoral regulation.. Blood.

[OCR_02154] PUCK T. T., MARCUS P. I. (1956). Action of x-rays on mammalian cells.. J Exp Med.

[OCR_02142] Patterson J. T., Pisano J. C., Di Luzio N. R. (1970). Reversal of antilymphocytic serum-induced immunosuppression by macrophage administration.. Proc Soc Exp Biol Med.

[OCR_02159] REVESZ L. (1958). Effect of lethally damaged tumor cells upon the development of admixed viable cells.. J Natl Cancer Inst.

[OCR_02169] Rickard K. A., Howard D., Morley A., Garrity M., Stohlman F. (1971). Stem cell stimulatory properties in vitro of an agar colony-stimulating.. Proc Soc Exp Biol Med.

[OCR_02176] Rickard K. A., Morley A., Howard D., Stohlman F. (1971). The in vitro colony-forming cell and the response to neutropenia.. Blood.

[OCR_02182] Salmon W. D., Hosse B. R. (1971). Stimulation of HeLa cell growth by a serum fraction with sulfation factor activity.. Proc Soc Exp Biol Med.

[OCR_02188] TABOR H., TABOR C. W. (1964). SPERMIDINE, SPERMINE, AND RELATED AMINES.. Pharmacol Rev.

[OCR_02195] Van den Brenk H. A., Moore V., Sharpington C. (1971). Growth of metastases from P-388 sarcoma in the rat followig whole body irradiation.. Br J Cancer.

[OCR_02207] Van den Brenk H. A., Sharpington C. (1971). Effect of local x-irradiation of a primary sarcoma in the rat on dissemination and growth of metastases: dose-response characteristics.. Br J Cancer.

[OCR_02219] Williams J. F., Till J. E. (1966). Formation of lung colonies by polyoma-transformed rat embryo cells.. J Natl Cancer Inst.

[OCR_02234] ZEIDMAN I., McCUTCHEON M., COMAN D. R. (1950). Factors affecting the number of tumor metastases; experiments with a transplantable mouse tumor.. Cancer Res.

[OCR_02199] van den Brenk H. A., Sparrow N., Moore V. (1969). Effect of x-radiation on salivary gland growth in the rat. I. Effect of single doses on post-natal differentiation and growth of acinar and duct components.. Int J Radiat Biol Relat Stud Phys Chem Med.

